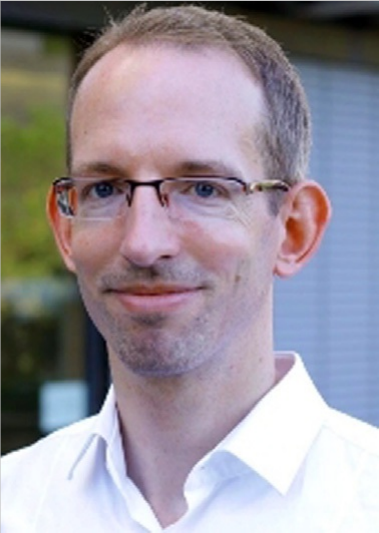# Prospects of Coupled Organic–Inorganic Nanostructures for Charge and Energy Transfer Applications

**DOI:** 10.1002/anie.201916402

**Published:** 2020-09-17

**Authors:** Anja Maria Steiner, Franziska Lissel, Andreas Fery, Jannika Lauth, Marcus Scheele

**Affiliations:** ^1^ Institute for Physical Chemistry and Polymer Physics Leibniz Institute of Polymer Research Hohe Str. 6 01069 Dresden Germany; ^2^ Institute of Macromolecular Chemistry Leibniz Institute of Polymer Research Hohe Str. 6 01069 Dresden Germany; ^3^ Technische Universität Dresden Mommsenstr. 4 01064 Dresden Germany; ^4^ Leibniz Universität Hannover Institute of Physical Chemistry and Electrochemistry Callinstr. 3A 30167 Hannover Germany; ^5^ Eberhard Karls-Universität Tübingen Institute of Physical and Theoretical Chemistry Auf der Morgenstelle 18 72076 Tübingen Germany

**Keywords:** inorganic nanostructures, optoelectronic Devices, organic π-Systems, plasmonics, self-assembly

## Abstract

We review the field of organic–inorganic nanocomposites with a focus on materials that exhibit a significant degree of electronic coupling across the hybrid interface. These nanocomposites undergo a variety of charge and energy transfer processes, enabling optoelectronic applications in devices which exploit singlet fission, triplet energy harvesting, photon upconversion or hot charge carrier transfer. We discuss the physical chemistry of the most common organic and inorganic components. Based on those we derive synthesis and assembly strategies and design criteria on material and device level with a focus on photovoltaics, spin memories or optical upconverters. We conclude that future research in the field should be directed towards an improved understanding of the binding motif and molecular orientation at the hybrid interface.

## Graphical abstract

This Review provides an overview of the field of organic–inorganic nanocomposites with a focus on materials that exhibit a significant degree of electronic coupling across the hybrid interface. The characteristic properties of these nanocomposites pave the way to potential optoelectronic applications in devices which exploit singlet fission, triplet energy harvesting, photon upconversion or hot charge carrier transfer.

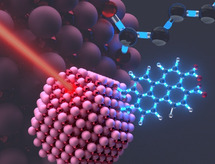

## 1 Introduction

With the ability to mix organic and inorganic compounds on the nanometer scale, the field of “hybrid organic‐inorganic nanostructures” evolved since the 1980s.^[1]^ The promise that such biphasic nanocomposites could exhibit emergent properties beyond the sum of the individual contributions from both phases due to a predominant effect of the interface has been the foundation of a dynamic field with continuously growing attention.[^2^, ^3^, ^4^, ^5^, ^6^, ^7^, ^8^, ^9^, ^10^, ^11^, ^12^] This large body of work may be bisected into hybrid nanocomposites with and without significant electronic coupling across the interface. While early examples like paints made from TiO_2_ particles dispersed in organic surfactants showed little electronic coupling across the interface, increasing attention is devoted towards coupled organic‐inorganic nanostructures (COIN), which we define as hybrid nanocomposites that transfer significant electron density or energy across the interface. In order for this to occur, both constituents of the composite are usually semiconductors, metals or conjugated organic π‐systems. As charge carriers or energy are exchanged, fundamental questions arise as to the direction, the efficiency and the speed of this transfer. Since exchanged charges carry magnetic momentum, another important question is the nature and fate of their spin in the process. The lifetime, dipole strength and degeneracy of excitons with different spin configurations are so distinct in inorganic vs. organic matter that the hybrid interface becomes a unique feature in any nanocomposite where the photophysical properties of both constituents merge into something potentially new. Studying charge and energy transfer in COINs exposes the effect of the interface, increases the chances that its role becomes predominant for the overall physical properties of the system, thus directly addressing the core hypothesis above. The four major applications to date of tailored charge or energy transfer in COINs are singlet fission, triplet energy harvesting, photon upconversion and hot electron transfer.[^6^, ^7^, ^8^, ^12^] Implementing these application schemes in optoelectronic devices bears the potential to improve the performance of solar cells, light‐emitting diodes, photodetectors and photocatalysis.[^3^, ^10^]

We argue here that advances in the chemistry of nanocomposites enable the rational design of solid‐state COIN films which explicitly target one of the four above‐mentioned major applications towards an improved optoelectronic device performance. A key factor is the ability to graft organic π‐systems directly to the surface of inorganic nanostructures, for instance by functional groups with strong electron donor/acceptor capabilities and thermodynamically favorable surface ligand exchange reactions.^[4]^ The advantages of this COIN assembly scheme over mere mixing are the suppression of phase segregation, a maximized number of hybrid interfaces and intimate contact between the organic and inorganic components.[^5^, ^13^] These advantages become especially apparent in the solid‐state, which is why we focus on solid‐state applications here. In addition, direct grafting allows controlling the macro‐/mesoscopic structure and long‐range order of the nanocrystals (NCs) in the composite via the nature of the organic surface molecules as well as the orbital overlap at the interface via the binding mode of the functional group.[^7^, ^14^] Other recent chemical advances are the synthesis of two‐dimensional nanostructures and lead halide perovskite nanocrystals, which introduce new optoelectronic functionalities into nanocomposites that were previously challenging to realize. Finally, we believe that the potential of core–shell nanocrystals has not nearly been exploited in COINs to the degree possible and appropriate based on the unique photophysical properties of these nanostructures.

### 1.1 Outline

This article is structured as follows: We begin with a short account of the most common inorganic (section 2) and organic (section 3) material classes from which COINs are typically formed. A special focus is the inorganic‐organic interface: After discussing the general role of surface ligands (section 3.1) we focus on π‐systems (section 3.2) and polymers (section 3.3) as ligands, and finally examine the coupling of conjugated organic systems to nanocrystals (section 3.4). In section 4, we detail the four main applications of charge and energy transfer across the hybrid interface in COINs, namely singlet fission, triplet harvesting, photon upconversion and hot electron transfer. For each application, we summarize the key criteria to be fulfilled by a COIN in order to excel in a specific application. In section 5, we outline tools for the characterization and fabrication of COIN‐based optoelectronic. This leads to the proposal of fabrication strategies in section 6 for COIN‐based devices geared towards photovoltaics, photodetection, spin memories and light emitting diodes. We conclude with an outlook on future challenges and directions for the field in section 7.

## 2 Inorganic Components

This section summarizes the most important inorganic nanostructures with which COINs have been reported up until now. While their general properties are described in the text, Table 1 lists specific examples of COINs previously applied for charge or energy transfer across the hybrid interface. The Table is presented with respect to the inorganic components; details of the organic components on the surfaces of these nanostructures are provided in section 3.

**Table 1:** Examples of inorganic nanostructures previously employed in hybrid nanocomposites with charge or energy transfer across the hybrid interface. (TIPS=Triisopropylsilyl.)

Inorganic component	Organic ligands and π‐systems
PbSe	Rubrene^[25]^ Pentacene^[26]^
	
PbS	5,11‐Bis(triethylsilylethynyl)anthradithiophene^[27]^ Rubrene (acceptor)^[28]^, 5‐carboxylic acid tetracene (transmitter)[^29^, ^30^] Tetracene^[31]^ 2‐Carboxylic acid TIPS‐tetracene^[32]^ 2‐Benzoic acid TIPS‐pentacene^[33]^ Zinc β‐tetraaminophthalocyanine^[13]^ Poly[sodium 2‐(2‐ethynyl‐4‐methoxyphenoxy)acetate]^[34]^
	
CdSe	Anthracene derivatives^[35]^ Pentacene derivatives^[36]^ Oligothiophenecarboxylic acid^[37]^ 1‐Perylenecarboxylic acid (PCA)^[38]^ 2‐Chloro‐9,10‐bisphenylethynylanthracene (acceptor), PCA (transmitter)^[39]^ Diphenylanthracene (acceptor), bis(pyridine) anthracene (transmitter)^[40]^ Diphenylanthracene (acceptor), 9‐anthracene carboxylic acid (transmitter)[^41^, ^42^] Zinc β‐tetraaminophthalocyanine^[43]^
	
CsPbBr_3_	PCA^[44]^ 1‐Naphthalenecarboxylic acid^[45]^ Diphenylanthracene (acceptor), aminodiphenylanthracene (transmitter)^[46]^
	
Two‐dimensional MoS_2_	Methyl orange, rhodamine 6G, methylene blue^[47]^ Zinc phthalocyanine^[48]^ Pentacene^[49]^
	
Au nanorods	Poly[2‐(3‐thienyl)‐ethyloxy‐4‐butylsulfonate] and poly(3,4‐ethylenedioxythiophene):polystyrene sulfonate^[50]^
	
Plasmonic CuSe_*y*_S_(1−*y*)_	Cobalt β‐tetraaminophthalocyanine^[51]^

### 2.1 Quantum dots

Quantum dots (QDs) are inorganic semiconductor crystallites which are confined in all three dimensions to length scales smaller than the exciton Bohr radius. Since most inorganic materials exhibit relatively large dielectric constants, strong dielectric screening is present in QDs. This invokes small exciton binding energies (10 s meV^[15]^), large exciton Bohr radii (up to 152 nm for PbTe QDs^[16]^) and the formation of Wannier‐Mott type excitons. QDs in the strong quantum confinement regime, that is, with radii much smaller than the exciton Bohr radius, exhibit drastically altered electronic structures compared to their bulk counterparts. Widened band gaps,^[17]^ discretization of electronic states close to the band edges^[18]^ and the occurrence of a phonon bottleneck,^[19]^ are just some of the remarkable properties arising in these materials. Another key property of QDs is relatively large spin‐orbit coupling.^[20]^ This, in conjunction with the electron‐hole exchange interaction and crystal field effects, leads to a fine‐splitting of the excitonic states with a different total angular momentum (*J*) of a few 10 meV (in II–VI materials) or even less (in III–V QDs).^[21]^ Since this is smaller than *k*_b_ *T* at not very low temperatures, QDs act as *spin mixers* with fast thermalization of spins.^[9]^ In many QDs, the excitonic state with the lowest energy is characterized by a total angular momentum of *J*=±2 and referred to as “dark”, manifesting in long lifetimes and low quantum yield at low temperature, when thermalization of spins is inhibited.^[22]^ This leads to an apparent analogy between singlet/triplet excitons in molecular emitters and bright/dark excitons in QDs with similar optical properties. However, this analogy is not universal as evidenced by lead halide perovskite nanocrystals.^[23]^ In these materials, the lowest excitonic state is a bright triplet exciton, manifesting in high quantum yield and relatively short lifetimes at low temperatures.^[24]^

To prevent surface reconstruction, the manifestation of defects and a deterioration of the electronic properties discussed above, the surfaces of QDs need to be passivated by some matrix.^[52]^ In epitaxially grown QDs, this is achieved by embedding the QDs in another inorganic material with matching lattice constant.^[21]^ In colloidal quantum dots, this passivation scheme is adopted in so‐called core–shell structures where the inorganic quantum dot core is passivated by a thin shell of another inorganic material with matching lattice constant. Depending on the band edge alignment between core and shell, these core–shell QDs are termed “type I” (a wide band‐gap shell encloses the narrow band gap core) or “type II” (core and shell are of comparable band gap in a staggered alignment).[^53^, ^54^] Type I architectures confine the exciton to the core and prevent the charge carriers from accessing surface traps. In contrast, type II architectures separate hole and electron spatially, which prolongs the exciton lifetime, promotes exciton dissociation and leads to red‐shifted emission.

The most common passivation scheme for colloidal ODs, however, is passivation by organic molecules tethered to the QD surface.^[55]^ Here, the interface between the QD and the surface ligand plays a central role in that the same nanostructure may exhibit a well passivated surface with one surface ligand species, while it suffers from frequent surface states upon passivation with another ligand species.^[56]^ Within Green's classification of ligands, the three most common classes of surface molecules for nanostructures are L, X, and Z‐type ligands, which differ in the formal number of electrons donated to the nanocrystal surface.^[57]^ For II‐VI nanocrystals such as CdSe, it has been shown that changing the number of Z‐type ligands (e.g. CdCl_2_) profoundly impacts the surface state density, while the number of X‐ (e.g. carboxylic acids) or L‐type ligands (e.g. amines) does not.^[58]^ Similar considerations seem to hold true for III–V QDs, and as a general paradigm the removal of excess surface anions by Lewis acids to heal surface states has been outlined.^[59]^ Since nanocrystals are facetted, preferred sites for ligand displacement exist, which are especially prone to defect formation and deserve particular consideration in limiting the defect density.^[60]^ Another source for surface trap states are metal‐based defects, which form either by the expulsion of a metal(0)‐species from the surface or the formation of metal‐metal dimers.^[61]^ To heal metal‐derived surface states, the beneficial effect of L‐type ligands has been suggested.^[61]^

In the context of this review, surface defects are also important as they often form a reservoir for excitons at the organic/inorganic interface before charge or energy transfer occurs. X‐type pentacene derivatives tethered to the surface of PbS quantum dots have been shown to induce defects, which localize triplet excitons at the surface of the quantum dots for 100 s ns, before the triplets are transferred to the organic π‐system.^[33]^ For lead halide perovskites coupled to 1‐pyrenecarboxylic acid (PCA), a large exciton probability density on the surface of the quantum dots was shown to be pivotal for efficient triplet transfer.^[44]^ This can be rationalized in terms of the well‐known dependence of Dexter energy transfer on large orbital overlap between the exciton donor and acceptor. Charge transfer from CdSe/CdS core–shell quantum dots to X‐type ferrocene derivatives proceeds in two short‐range charge transfer steps via a reversible, shallow trap state reservoir at the quantum dot surface.^[62]^ It can be argued that the reversibility and short‐range transfer mechanism increases the overall transfer probability with respect to a direct, single transfer. Finally, Rubrene tethered to PbS quantum dots showed a three orders of magnitude increase in photon upconversion yield via triplet‐triplet annihilation upon intentionally introducing mid gap trap states at the surface via cationic adsorbates.^[28]^ Such findings not only highlight the importance of defects for charge and energy transfer across the hybrid interface but also trigger the question about the spin character of dangling bonds and surface states in quantum dots. In the aforementioned works, this is believed to be sufficiently triplet‐like, and the angular momentum during exciton transfer should be conserved.

### 2.2 2D Nanostructures

**Figure 1:**
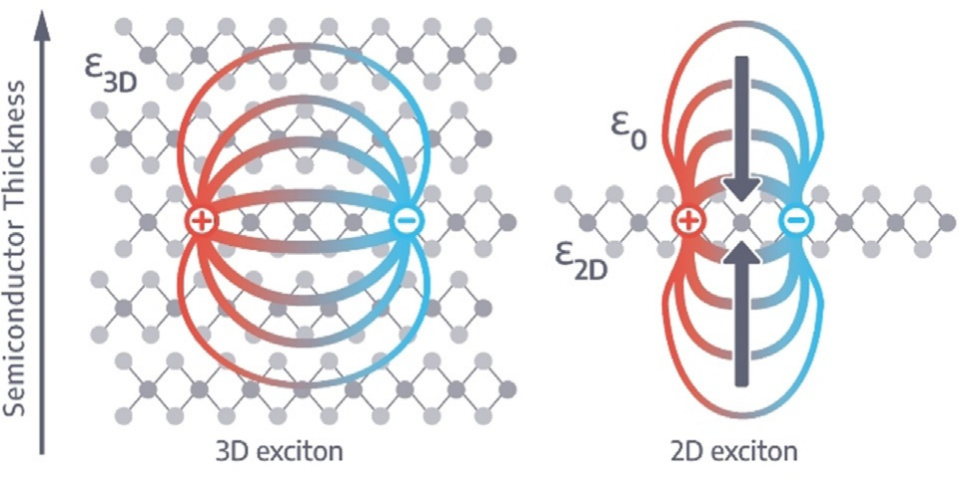
Electronic and excitonic dimensionality effects in 2D semiconductors. Bound excitons exhibit decreased dielectric screening and increased Coulomb interaction in the 2D semiconductor compared to the solid state.^[65]^

The rise of two‐dimensional (2D) materials with outstanding electronic properties has started with the ongoing research on graphene 15 years ago, and in recent years ultrathin 2D semiconducting materials with well‐defined band gaps have likewise moved into focus.^[63]^ These 2D semiconductors are highly promising for innovative (opto)electronics including fast‐switching transistors, efficient light‐emitting diodes and regenerative energy conversion applications.^[64]^ They exhibit, additionally to a band gap that is dependent on their thickness, significantly increased (bi)exciton binding energies in comparison to their solid‐state and quantum dot counterparts.^[65]^ This originates from reduced dielectric screening with decreasing thickness of the materials and increased Coulomb interaction of charge carriers (electrons and holes) in the structures (see Figure 1). Exciton binding energies in atomically thin 2D transition metal dichalcogenides MX_2_ (TMDC, M=W, Mo; X=S, Se) have values in the range of ≈300–600 meV so that excitonic features are prominent at room temperature.[^65^, ^66^]

While deposition and exfoliation routes for the production of layered 2D van der Waals semiconductors are widely used, colloidal synthesis methods for obtaining ultrathin 2D semiconductors have opened up a promising alternative to the classical exfoliation and deposition methods.[^67^, ^68^] By using wet chemistry methods known from colloidal nanocrystal synthesis, the thickness of ultrathin 2D semiconductors can be tailored to atomic precision, leading to the control and fine‐tuning of the desired optoelectronic properties induced by the (strong) quantum confinement in the out‐of‐plane direction. Depending on their thickness, colloidal (ultrathin) 2D semiconductors thus feature strongly bound excitons and/or highly mobile free charges.^[69]^ A key advantage of colloidal chemistry methods is the ability to grow intrinsically isotropic materials (e.g. cubic lead chalcogenides) into a strongly anisotropic crystal shape by the virtue of surface ligands and to control the reaction conditions and kinetics.[^68^, ^70^] The interaction of organic π‐systems (organic semiconductors) with the flat geometry of 2D semiconductors is highly interesting and leads to efficient electronic and structural coupling.^[11]^ The flat geometry and anisotropy of thin 2D semiconductors offers a new degree of freedom for combining properties that neither of the single components can feature alone.

### 2.3 Plasmonic nanocrystals

Metal nanostructures possess excellent light harvesting properties, due to the phenomenon of localized surface plasmon resonance (LSPR).^[71]^ LSPR occurs when electromagnetic radiation excites a resonant, collective oscillation of the quasi‐free conduction electrons within the nanoparticle. This oscillation is characterized by an increased extinction coefficient at the resonant frequency.^[72]^ In addition, the spatial restriction of the resonance allows the confinement of incident light beyond the diffraction limit, resulting in a localization and enhancement of electromagnetic (EM) fields on the sub‐wavelength scale.^[73]^ The optical response of the individual nanoparticle can be tuned over a wide range of wavelengths by variation of the particle size and shape, the composition, and/ or the refractive index of the environment.[^72^, ^74^] For example, anisotropic nanoparticles, such as nanorods, exhibit different plasmon modes than spherical particles. Thus, the introduction of anisotropy enables the extension of the accessible resonance wavelengths. Furthermore, particles with anisotropic geometry exhibit an increased polarizability and a stronger EM field enhancement on regions of high curvature.[^75^, ^76^]

Compared to lithographic fabrication methods, wet‐chemical synthesis offers a cost‐efficient and scalable way to produce defined nanocrystals.^[77]^ To date, a multitude of plasmonic nanocrystals consisting of different sizes, morphology, or material has been established by the wet‐chemical approach.^[74]^ In particular, the seed‐mediated growth method has been proven to yield particles with a small size distribution and variety of available shapes.^[78]^

Besides tuning at the single‐particle level, the plasmonic coupling of nanoparticles provides an additional way to further enhance and control these effects. The plasmonic coupling leads to new emerging properties such as strong electromagnetic field enhancement,^[79]^ hybridized plasmon modes,^[80]^ and a strong optical response when varying the distance between coupled nanoparticles.^[81]^ As an example, the local field enhancement between nanoparticles can be used to achieve a higher sensitivity in Raman scattering spectroscopy.^[82]^

Due to the aforementioned properties, plasmonic nanocrystals facilitate a variety of applications including electronics (e.g. plasmonic solar cells),^[83]^ optics (e.g. overcoming the diffraction limit)^[73]^ or sensing (signal/sensitivity enhancement).^[84]^

## 3 Organic Components

**Figure 2:**
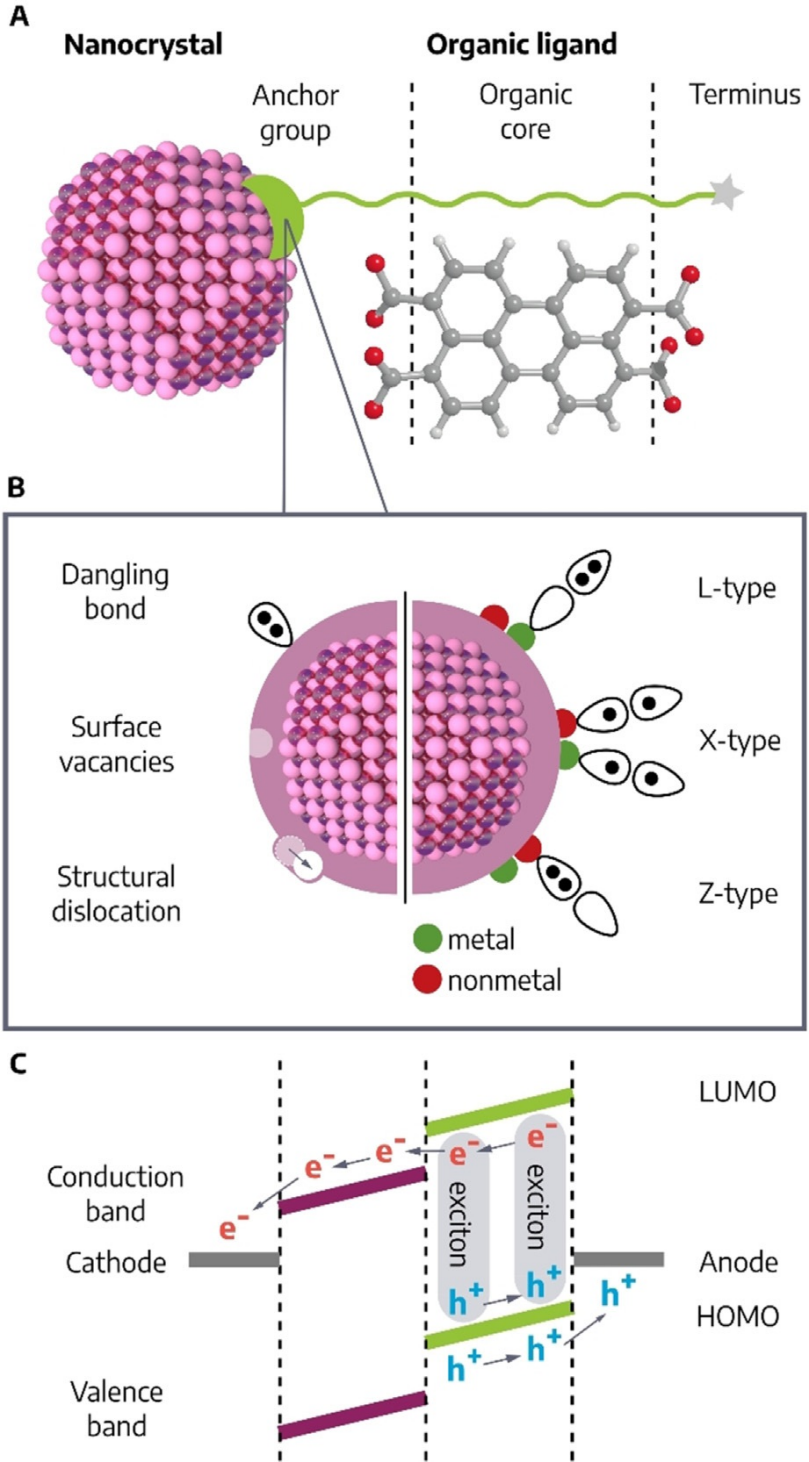
A) Schematics of the NC/ligand hybrid interface. B) Schematics of the surface of a nanostructure with the three most common surface defects on the left. The right side illustrates the three most common types of organic anchor groups according to Green's classification^[57]^ and their interaction with dangling bonds on the surface of the nanostructure. C) Schematic of an NC‐organic solar cell. A photon causes exciton formation in the organic ligand, the hole flows through the organic (green) HOMO/valence band towards the anode, and the electron to the NC (purple) and finally cathode.

This section focuses on the qualities of the organic ligands found in COINs. It will discuss the general nomenclature of the ligand shell (see Figure 2), and briefly describe π‐conjugated organic and polymeric ligand systems ‐ a selection of specific examples is given in Table 1. Emphasis is on the mechanical and electronic coupling of inorganic NCs and organic ligands, which is central for the stability as well as for the optoelectronic properties of the systems.

### 3.1 Organic surface ligands

Organic surface ligands (see ref. [85] for a recent comprehensive review) are essential to provide solubility during wet‐chemical nanoparticle growth,^[86]^ and to maintain colloidal stability by minimizing the NC surface energy, either via electrostatic repulsion or steric effects. Which ligands are needed and suitable depends on the NC material, the targeted application and environment (e.g. solvent polarity, pH). Ligands define the accessible NC size:^[87]^ Thiols for example limit Au NC growth below 10 nm,^[88]^ while in the presence of citrates metallic NCs can be grown to sizes above 100 nm.^[87]^ Also, by interacting differently with NC facets, ligands can be shape directing and enable harvesting anisotropic NCs, for example, nanorods, cubes, and stars:^[89]^ Amphiphilic cetyltrimethylammonium bromide (CTAB) for example binds preferentially to the (100) plane, promoting an anisotropic growth on the (111) crystal facets.^[90]^

The structure of surface ligands can be formally divided in (1) anchor group, (2) ligand core and (3) termini (see Figure 2). The anchor group is the point of contact between ligand and NC, and can be either an intrinsic part of the ligand (e.g. the imine group of polyaniline)^[91]^ or a distinct functional group introduced to enable binding (e.g. amino groups on a phthalocyanine). The anchor group must have a high affinity to the atoms comprising the NC. Typical anchor groups for metallic NCs are thiols (R‐SH), amines (R‐NR_2_), carboxyls (R‐COOH), and phosphines (PR_3_); and for semiconducting QDs thiols, carboxyls, phosphine oxide (P(O)R_3_), and phosphonyls (PO(OR)_2_). Several general rules apply: The interaction between the particle surface and the anchor group can be ionic or covalent and can have different degrees of dipolar (dative) character. Green's classification distinguishes X‐type ligands with a strong covalent character (e.g. thiols on CdSe), L‐type ligands bonding in a donor‐acceptor fashion via a lone pair donated by the anchor group (e.g. Au‐NR_2_), and Z‐type ligands accepting electron pairs from the surface (e.g. CdCl_2_).^[57]^ Many properties of the NC‐ligand bond can be explained by Pearson's HSAB (hard/soft acid/base) concept, for example, the higher affinity of Se‐based anchors compared to S‐based ones correlates to the softness of Au.^[92]^ Especially charged anchor groups with a high coordinative character (e.g. carboxyls) are susceptible to changes in pH and salt concentration. While multidentate as well as multipodal binding schemes strengthen the mechanical binding via chelate effects, especially multipodal anchors often contain conjugation breakers such as sp^3^ hybridized carbon atoms^[93]^ and higher denticities,^[94]^ such as macrocyclic layouts are comparably rare.^[95]^

Ligands are either already present during wet‐chemical NC growth or introduced at a later stage via exchange reactions (post‐modification). In general, to replace the ligands already present on the surface, the anchor group of the incoming ligands needs to have a higher affinity and bind more strongly to the surface, yet different synthetic strategies can be used to overcome this rule, e.g using sacrificial intermediate ligands in a two‐step protocol. The binding affinity of diethylamine (DEA) is pH‐switchable: The native amine is a strong ligand, while the protonated form interacts only weakly with the gold surface. This allows to replace strong surface ligands using the amine, before exchanging the protonated form with a weak ligand in a second step.^[96]^ Regarding one‐step ligand exchange reactions, thiols replace carboxyls on gold due to higher adsorption energy, while amines with a similar energy were experimentally shown to overcoat rather than displace a citrate layer.^[97]^ When using the binding energy as an indicator of the probability of bond formation, thiophene (T) derivatives, pyridine (Py), and thiols have a high probability to form bonds to metal surfaces compared to for example, amines.^[98]^ The affinity of cyano (CN) ligands to gold is predicted to be high, yet in experiments CN has often been found to be a weak anchor group with low binding probability.[^92^, ^98^] Considering ligand exchange on gold NCs synthesized with citrate ligands, in situ monitoring by SERS showed that thiolates and bovine serum albumine (BSA) were more efficient than carboxylates and inorganic chloride due to the higher binding energies with the Au NC surface.^[97]^ The surface of CdSe QDs is defined by X‐type ligands binding to excess cadmium ions, and ligand exchange reactions have to balance the surface charge, for example, by substituting with chloride or thiols.^[99]^ Anchor groups define the electronic coupling between NC and organic ligand (see 3.4) and are therefore a key component in the design of plasmonic NPs. While constantly developing, research is still centered on classic anchor groups. Looking towards the area of unimolecular electronics might be interesting for further research:^[100]^ Covalent bonds formed by sp‐hybridized carbons are exceptionally stable and enable a strong hybridization of molecular and metal states.[^92^, ^101^, ^102^] Recently, N‐heterocyclic carbenes emerged as promising new ligands on gold surfaces, as they exhibit a much‐enhanced stability compared with for example, thiols.^[103]^ Based on DFT‐based calculations of the NHC−Au bond, the electron density is delocalized in the HOMO over the gold and carbene carbon to the nitrogen atoms,^[104]^ and furthermore, the highly covalent NHC linkages were shown to reduce the work function of the metal.^[105]^ Despite this, studies on NHC‐coupled systems of NCs and organic π‐systems are still missing.

The ligand core determines the properties of the organic ligand. Saturated chains, for example, alkyl or ethylene glycol chains, are primarily used for solvation and colloidal stabilization. As these chains are insulating, interaction between NCs depends on the length of the ligand core, and shorter cores generally lead to stronger coupling.^[106]^ Hybrid systems of inorganic NCs and electronically functional conjugated ligands can have multiple functions beyond the sum of the single components due to the strong synergies created at the interface.

Finally, the ligand termini can be used as reactive sites, for example, to tune the interaction of NCs with cells or functional surfaces, or to modify the hydrophilicity of the colloidal NPs.

### 3.2 π‐Systems

Organic semiconductors are electroactive themselves and their low dielectric constants lead to the formation of strongly bound Frenkel type excitons. NCs can serve as sensitizers for molecular triplet states via triplet energy transfer processes, or alternatively, the triplet exciton is transferred from the organic π‐system to the inorganic NP, for example, from tetracene to a PbS NC.^[31]^ In the latter case, the triplets are generated in the tetracene via singlet fission (see 4.2), which rapidly produces dark triplet excitons (*τ*<200 ps) with yields approaching 200 %. Both transfer directions are Dexter‐type, requiring orbital overlap and thus exhibiting an exponential distance dependence.[^25^, ^26^]

Efficient exciton dissociation and charge separation in NC‐organic hybrid materials requires an alignment of the positions of the frontier orbitals of the organic π‐system and the electronic states available at the NC surface: For the combination of an organic donor and a NC acceptor, the NC′s LUMO level must be below the LUMO level of the organic ligand (see Figure 2). Two classes of conjugated systems can be distinguished: Defined monomeric or oligomeric organic dyes, and conjugated polymers (CPs). Commonly used small molecule organic dyes are polyacenes, for example, rubrene^[25]^ and anthracene derivatives,^[39]^ phthalocyanines^[13]^ and oligothiophenes (see Table 1).^[37]^

Flat and conjugated organic dyes, for example, phthalocyanines, can stack into aggregates, altering the molecular properties of the π‐system significantly (see ref. [107] for an in‐depth discussion of the stacking): In J‐aggregates, the absorption band is bathochromically (to longer wavelengths) shifted compared to the free monomer, and a nearly resonant fluorescence (i.e. very small Stokes shift) with a narrow band is observed. In H‐aggregates, the absorption band is shifted to shorter wavelengths (hypsochromically) compared to the monomer, and low or no fluorescence is observed. Blended composites of thiacyanine J‐aggregates and ZnCdS(CdSe) NCs show a 2.5‐fold increase in photoluminescence intensity compared to pure NCs, with a 90 % energy transfer efficiency.^[108]^ This can be boosted further to 98 % and 5‐fold increase when the NCs are carrying a conjugated organic ligand able to interact with the J‐aggregate.^[109]^

### 3.3 Polymers

Non‐conjugated polymers of defined lengths can be used to build‐up ordered 2D and 3D lattices with controlled NC distances and geometries.^[14]^ The combination of NCs and conjugated polymers (CPs) is of special interest to obtain functional hybrid materials. The electronic and spectroscopic properties of CPs are determined by the energetic positions of HOMO and LUMO, which are influenced by the polymer's regioregularity, polydispersity, and average molecular weight for a given system.^[110]^

Composites formed from CPs and bare NCs phase segregate,^[5]^ thereby forming large interface areas for charge separation and leading to improved quantum efficiencies in photovoltaic devices.^[111]^ The embedment of NCs carrying alkylated ligands into a polymer matrix similarly leads to phase segregation, as the ligands prevent the incorporation of NCs into the crystallites. So‐called hairy particles are true hybrids of chemically bound NCs and CPs, formed by grafting polymer chains to the surface via ligand exchange, or by grafting CPs from the NC surface using a chain growth mechanism.^[112]^ For the grafting‐to method, CPs need to carry suitable anchor groups to replace weaker ligands present on the surface, for example, a polythiophene end‐capped with amine was bound to CdSe nanorods carrying pyridine to give homogeneous films of the hybrid material.^[113]^ The grafting‐from polymerization requires a suitable initiator, and NCs covered in low molecular weight initiators can suffer from reduced colloidal stabilization. Also, the bulky transition metal catalyst systems commonly used in CP chain‐growth polymerizations compete for space on the surface, and especially for larger NCs with a lower surface curvature sterical hindrance must be taken into account.^[114]^ Another interesting route is to use molecular recognition to couple CPs and NPs, for example, the capping of CdSe with thymine ligands tethered to the surface allowed the binding of polythiophenes carrying diaminopyrimidine‐functionalized sidechains via hydrogen‐bonds,^[115]^ which are prototypical mechanophores enabling reversible and responsive binding. This promising approach also exemplifies a continuing challenge: To combine a mechanically robust binding in CP‐NC hybrids with strong electronic coupling is not trivial, as the CP is often bound via an anchor group not enabling charge delocalization over the organic‐inorganic interface. Also, for metallic NCs, only few NC‐CP hybrids are described, and in these, the NCs are usually immobilized or embedded within a CP matrix without defined electronic coupling across the inorganic‐organic interphase.

### 3.4 Coupling of organic π‐systems to nanocrystals

Due to the high surface‐to‐volume ratio, the surface plays a dominating role in NCs, to the point of introducing functionalities such as surface plasmon resonance or catalytic activity. Surface vacancies, reconstruction events and dangling bonds can introduce new electronic states.^[116]^ In general, shallow traps originate from surface disorder and reconstructions, deep traps are associated to low coordinated atoms on the surface: Surface atoms of NCs have weaker bonds compared to bulk atoms, creating electronic states able to trap photoexcited charges before they can go through radiative recombination. Organic ligands binding to the surface can passivate these states to enable luminescence. They saturate dangling bonds, screen the NC from its environment, and the interaction with functional (e.g. π‐conjugated) organic ligands can alter the energy, optical performance and reactivity of an NC surface.^[117]^

When forming a contact between an NC and an organic molecule, both the mechanical and electronic coupling have to be taken into account. The mechanical coupling defines the probability of forming a contact and the stability of the resulting hybrid structure, whereas the electronic coupling describes the overlap between the frontier orbitals of the organic molecule and the electronic states available at the particle surface, and is the key parameter for charge injection from the metal to molecule and vice versa. When regarding the inorganic and organic parts as a donor‐acceptor system and the anchor group as bridging ligand, parallels can be drawn with Taube's concept of inner and outer sphere electron transfer:^[118]^ To achieve a highly coupled through‐bond inner sphere transfer, a non‐innocent ligand is required. This can either be an atomic ligand^[119]^ or a conjugated group, preferably with covalent character.^[102]^ Outer sphere electron transfer on the other hand occurs through space, and is dependent on spatial proximity and driving force, that is, potential difference,^[120]^ thereby accounting for the length dependence in NC systems coupled by insulating ligands.^[106]^

## 4 Optoelectronic Effects in Coupled Organic–Inorganic Nanostructures and Prospects for Applications

**Figure 3:**
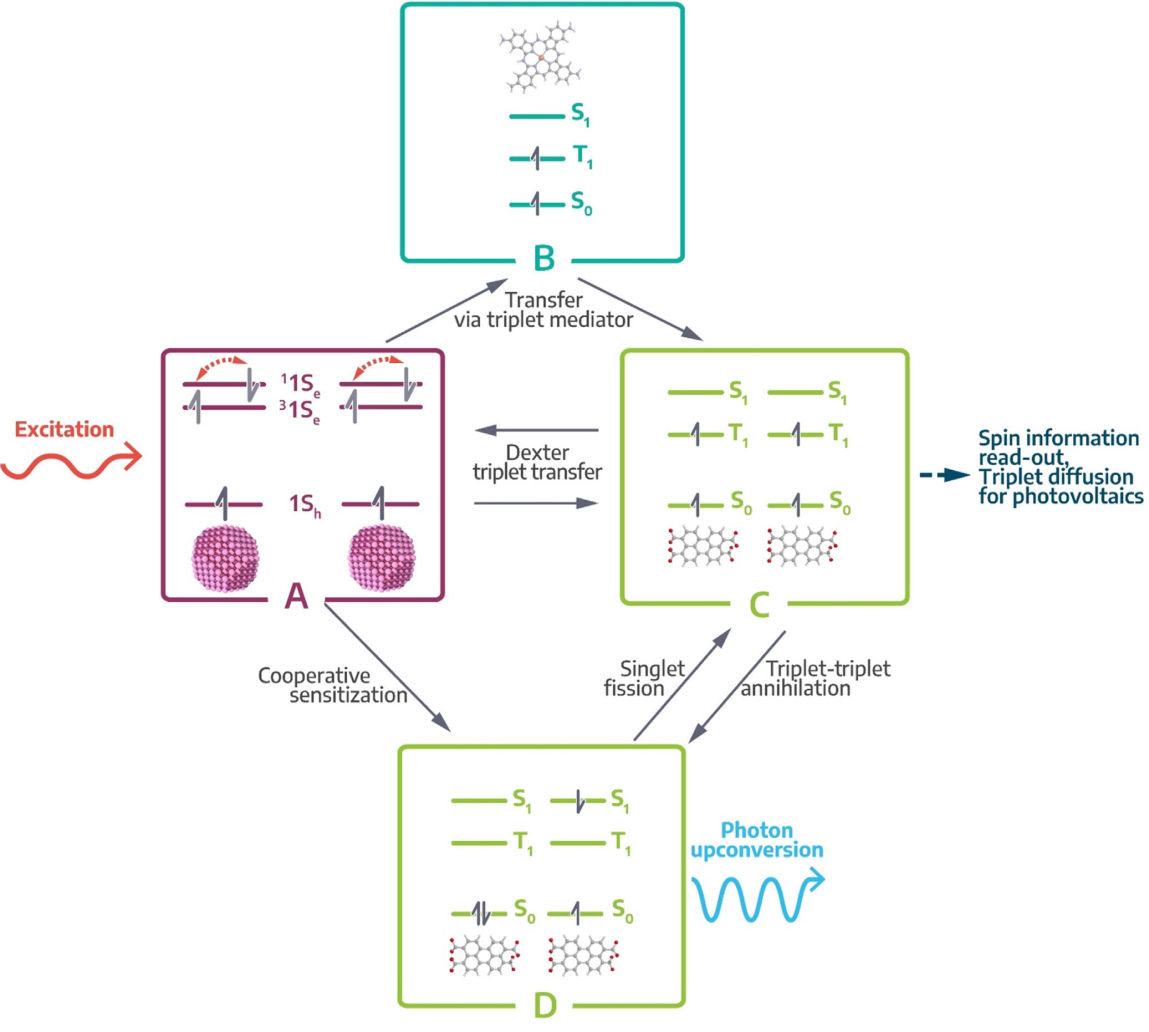
Relationship between singlet fission, triplet harvesting and photon upconversion in COINs with an inorganic sensitizer and organic acceptor. A) Optical excitation of the inorganic nanostructure affords spin‐mixed excitons with a high fraction of triplet spins. Dexter energy transfer, either indirectly via a triplet transmitter B) or directly across the hybrid interface, enables triplet harvesting by the organic component in C). From here, triplets can either be used in devices or undergo triplet‐triplet annihilation to yield the high‐energy singlet in D). Alternatively, this singlet is also afforded directly by cooperative sensitization from the singlet fraction in A). The singlet can either be subject to fission to restore the two low‐energy triplet states or relax radiatively to the ground‐state by emitting a photon of higher energy than the initial photon used during excitation. The latter is called photon upconversion.

In this section, we describe the four fundamental processes in COINs arising upon photoexcitation and consecutive charge/ energy transfer. While the similarities and interrelations of the four processes are schematically summarized in Figure 3, each subsection lists the specific details and concludes with an account of the distinct requirements to optimize the particular process for efficient application in devices.

### 4.1 Singlet fission

In singlet fission (SF), a chromophore in an excited singlet state S1 shares its excitation energy with a neighboring ground‐state chromophore, resulting in the conversion of both into excited triplet states T1 (Figure 3, pathway D‐C).^[121]^ These are initially coupled into a doubly excited pair of spin‐correlated triplets considered a dark state, before the formation of two fully independent triplet states. SF is spin‐allowed and can therefore proceed at ultra‐fast rates and sub‐100 fs timescales.^[122]^ Different SF mechanisms are discussed, and for SF to be efficient, it needs to be fast to outcompete other photophysical processes, for example, (non‐)radiative recombination, excimer formation, and charge and energy transfer at heterojunctions.[^121^, ^123^]

First described in 1965, accounts mostly focus on SF events between two organic components in solid state as well as in solution. Only recently the organic‐inorganic interface is being more intensely studied due to the potential of SF as an organic‐centered multiple exciton generation process to overcome the theoretical limit for solar cell performance described by Shockley and Queisser: In a singlet fission photon multiplier (SF‐PM), each high‐energy photon absorbed by the organic SF component would generate two triplet excitons, which would then be converted to two low energy photons and absorbed by a conventional PV cell, doubling photocurrent harnessed from the high‐energy part of the solar spectrum. SF‐PMs could break the Shockley‐Queisser limit for the silicon band gap and increase the efficiency of the best Si PV cells available today from 26.7 % to 32.5 %.^[124]^

Still, SF requires a suitable organic chromophore, and the number of molecule classes known to meet the fundamental requirement that the singlet excitation energy *E*(S1) is higher than ‐ and yet well matched to ‐ the sum of the two triplet energies *E*(T1) is limited:^[121]^ Most notable systems include acenes, carotenoids, diphenylisobenzofurans, and rylenes. Seeking to expand this library, Troisi et al. used quantum chemical calculations to screen for potential SF candidates among known compounds, but the success rate of such predictions is yet to be experimentally determined.^[125]^

Disregarding the case of an all‐organic process taking place at an inorganic surface, and focusing on SF events across the interface, two directions of energy transfer are possible: From the organic component to the inorganic, and vice versa. In the first case, organic chromophores absorb light to yield a singlet state, and after SF the resulting triplets are transferred to NCs, which in turn emit two photons. Combining pentacene with PbSe NCs of different sizes (and band‐gaps) allowed to pinpoint the triplet energy of the chromophore between 0.85 eV and 1.00 eV in operating devices.^[126]^ Studies of thin bilayer samples of PbSe NCs and pentacene demonstrate that triplet energy transfer (TET) from the SF chromophore takes place only when the NC band gap is resonant with the molecular triplet energy.^[26]^ In a MAPbI_3_/TIPS‐pentacene bilayer system, SF generated triplet states of the pentacene can be transferred to the conduction band of the perovskite.^[127]^ Rao et al. used TIPS‐tetracene as a solution‐based SF active chromophore: After an efficient SF process in solution, TET to PbSe NCs takes places, with surface‐associated tetracene ligands key to mediate an efficient transfer.^[128]^ Similarly, SF and TET take place when the chromophore is directly bound to the PbSe NC.^[129]^

From a fundamental point of view, the NC to organic transfer direction is especially interesting: The NC band gap can be tuned, and even energetically high‐lying naphthalene triplets are accessible via visible‐light‐driven TET from CsPbBr_3_ NCs.^[45]^ To utilize the high absorption coefficient of NCs for SF, Beard et al. coupled triisopropylsilylethynyl pentacene carboxylic acid to CsPbBr_3_ NCs:^[130]^ Upon photoexcitation of the NCs, a fast hole transfer to the pentacene occurred, followed by an electron transfer process generating an excited singlet state, and subsequent SF event of the organic component, with a triplet lifetime of around 10–14 μs and intrinsic triplet quantum yield (Φ_SF_) of 145 %.

### 4.2 Triplet exciton harvesting

Triplet excitons are referred to as “dark excitons” as selection rules require the simultaneous flip of one carrier's spin during recombination, which is unlikely, and results in very low emission yields.^[131]^ Different from singlets, there is no intrinsic limit to the diffusion lengths of triplets in thin films, and in theory, triplets can travel over very long distances (several μm).^[132]^ Due to strong exchange interactions, triplets in organic π‐systems carry substantially less energy than the corresponding singlet exciton, so that spontaneous triplet‐to‐singlet conversion is rare and the energy stored in triplet states is often lost for further applications, such as LEDs. On the other hand, the triplet lifetime in these materials can be of the order of milliseconds, which is desirable for spin memory applications. Thus, most organic π‐systems are poor triplet sensitizers but good triplet acceptors for long‐term storage. In contrast and as detailed in section 2.1, QDs act as *spin mixers* at room temperature with fast thermalization of spins, which renders them good triplet sensitizers but poor acceptors with low spin stability.

Following these general considerations, combining QD‐based triplet sensitizers and organic π‐systems for the long‐lived storage of excitons with triplet spin into COINs enable exciting new possibilities for optoelectronics (Figure 3, pathway A–C). For instance, control over the spin properties in excitonic materials is of pivotal importance for future applications as quantum objects, where a single charge carrier in an isolated quantum object may serve as a qbit ‐ the fundamental unit of information in a quantum computer ‐ if its spin can be controlled and preserved.^[21]^ Furthermore, triplet harvesting and fast triplet transfer is often the rate‐limiting step in multiexciton generation by singlet fission (see section 4.1), which bears the potential to exceed the Shockley‐Queisser efficiency limit for solar cells.^[128]^ Material combinations tested in this regard so far are summarized in Table 1.[^25^, ^27^, ^35^, ^36^, ^37^, ^38^, ^44^, ^45^] A crucial bottleneck for triplet transfer is the competing recombination of the triplet exciton to the NC ground state via intersystem crossing, which often occurs on a nanosecond timescale, that is, much faster than in organic π‐systems. For this reason, the introduction of a triplet transmitter between the surface of the NC and the triplet acceptor may become necessary (Figure 3, pathway A‐B‐C). If triplet transfer from the NC to the transmitter is faster than the excitonic lifetime in the NC and consecutive transfer from the transmitter to the acceptor is thermodynamically favorable, the overall efficiency of triplet transfer increases drastically. Table 1 lists some examples for transmitter‐enabled triplet transfer.[^29^, ^30^, ^39^, ^40^, ^41^, ^42^, ^46^]

If the triplet transfer kinetics from the NC to the organic acceptor is unfavorable vs. ground state relaxation, exploring the opposite direction may also be rewarding (Figure 3, pathway C‐A). Using organic π‐systems such as pentacene, tetracene or 2‐carboxy(TIPS)tetracene as the triplet sensitizer and PbS or CdSe NCs as the acceptor has been demonstrated to enable very efficient triplet harvesting.[^26^, ^31^, ^32^, ^38^] The advantage of these systems is that much longer triplet transfer times are tolerated due to the long triplet lifetime in the organic sensitizers of up to 10 milliseconds and that triplets are readily accepted by the NCs due to their inherent spin tolerance.

A noteworthy combination of the two aforementioned triplet harvesting pathways is “reverse triplet energy transfer”, which occurs if both pathways are comparably fast. This process, which should be generally feasible at any organic π‐system/NC interface with near‐resonant alignment of triplet states, is a powerful tool to engineer NC‐based fluorophores with extremely long lifetimes on the order of 100 s microseconds.^[38]^

The efficiency of triplet exciton harvesting can be viewed roughly as the product of the efficiency of triplet generation in the sensitizer and the efficiency of TET to the acceptor (optionally via a transmitter). With QDs as the sensitizer, intersystem crossing occurs so readily that triplet generation is usually not the rate limiting step. For efficient triplet harvesting to occur, the exciton recombination rate in the QD needs to be slower than the rate of TET. This can be ensured by passivation of deep trap states, for instance by one of the core–shell structures outlined in section 2.1. We suggest that the utilization of type‐II core/shell QDs as the sensitizer is particularly promising due to the greatly prolonged exciton lifetimes.^[54]^ It has thus been argued that the main challenge in optimizing triplet exciton harvesting is increasing the efficiency of TET, and designing strategies for tailored ligand molecules for QDs.^[128]^ In contrast, shallow surface states of QDs can even be beneficial during TET by acting as a temporary storage place for triplets before they are transferred to the acceptor.^[33]^

Due to their low transition dipole moment, TET between the sensitizer and the acceptor is dominated by the Dexter mechanism, where two individual charges are consecutively transferred and spin is exchanged. According to Marcus theory, the Dexter rate for this exchange is given as [Eq. 1]:

(1) $$\tau_{TET}^{-1}=\;\frac{\epsilon^{2}}{\hbar }\sqrt{\frac{\pi }{k_{B}T\lambda }}exp\left(-\frac{{\left(\lambda +\Delta V\right)}^{2}}{{4\lambda k}_{B}T}\right)$$

Here, *ϵ* is the coupling energy between the sensitizer and the acceptor, *λ* is the Marcus’ reorganization energy and Δ*V* is the energy offset between the triplet state of the sensitizer vs. that of the acceptor. In the weak coupling regime (that is, if the tunneling probability Γ≪1), the coupling energy may be approximated as [Eq. 2]

(2) $$\epsilon =\;\frac{\Delta V}{\pi }exp\left(-2\frac{\sqrt{2m^{{}^{*}}\Delta V}}{\hbar }d\right)$$

where m* is the effective mass of the transferred charge and *d* is the distance between the sensitizer and the acceptor.^[133]^ This, somewhat idealized, quantification of the efficiency of triplet exciton harvesting implies that future strategies for the design of coupled organic‐inorganic nanostructures for triplet harvesting should concentrate on accomplishing a large negative energy offset Δ*V*, and decreasing m* of the transferred charges, *λ* as well as *d*.^[134]^ The latter arguments are largely reflected in the crucial dependence of efficient Dexter transfer on ample orbital overlap between the sensitizer and acceptor.^[135]^ Furthermore, long‐range order and orientation of sensitizers as well as acceptors is crucial for maintaining long triplet lifetimes and enabling triplet diffusion.^[132]^

We arrive at the following key properties for COINs to be fulfilled for efficient triplet exciton harvesting:

For the sensitizer, these are long exciton lifetimes, high lying triplet states, a large extinction coefficient and long‐range order in thin films. For the acceptor, a low‐lying triplet state, strong binding affinity to the sensitizer with a rigid binding motif, for example, by multidenticity, short binding distance as well as long‐range order and iso‐orientation are required.

### 4.3 Photon upconversion

**Figure 4:**
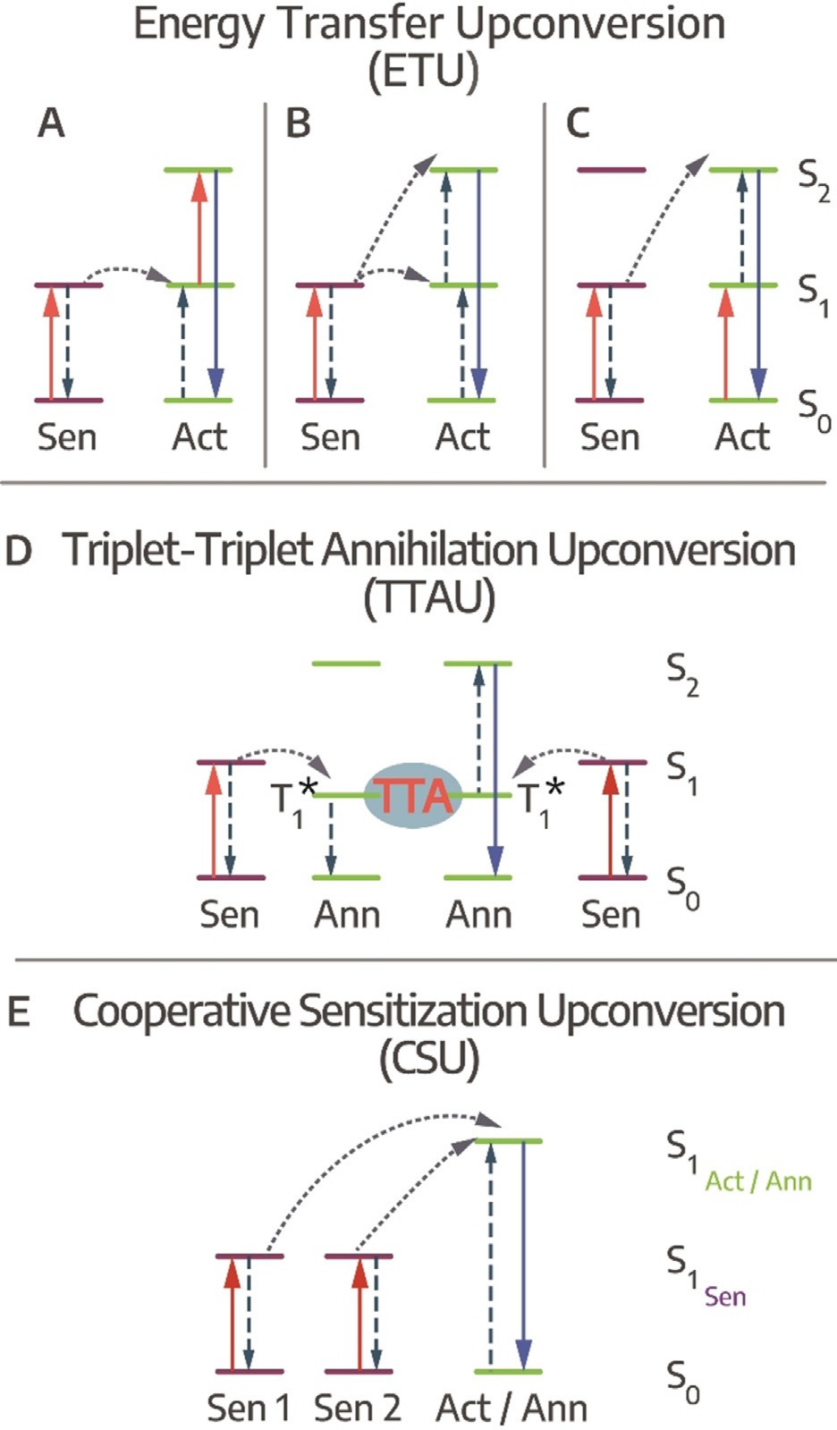
Upconversion pathways, ETU via A) ET from directly excited sensitizer and following excited state absorption and PU, B) two successive ET from directly excited sensitizer lead to PU, C) cross‐relaxation PU of two similar sensitizer/ activator units, D) TTAU from photoexcited sensitizers undergoing TET to yield PU via TTA, E) CSU involves at least two sensitizer units undergoing PU via two cooperatively interacting sensitizers.

Photon upconversion (PU) is a nonlinear process occurring at low photoexcitation densities in which multiple (at least two) photons of lower energy (longer wavelength) are sequentially converted into a photon of higher energy (shorter wavelength), which is apparent by Anti‐Stokes emission from the sample.[^136^, ^137^] In this work, we will focus on sequential PU, excluding two‐photon absorption, second‐harmonic generation (SHG) and direct excited state absorption upconverting processes. While PU has been studied in rare earth element‐based solid‐state (NC) materials since the 1960s,[^137^, ^138^] here we will address PU processes in COIN systems, where the combination of organic and inorganic semiconductor nanostructures via a coupled interface is needed for efficient upconversion. Typical NC systems coupled to a variety of organic molecules can be found in Table 1. PU in COIN can be described as a process between two components consisting of the directly photoexcited *sensitizing* system absorbing the lower energy photons and the *activator/annihilator* system from which anti‐Stokes emission takes place. For radiative recombination PU discussed here, three general methods are possible (Figure 4):

1. Energy transfer upconversion (ETU)
2. Triplet‐triplet annihilation upconversion (TTAU)
3. Cooperative sensitization upconversion (CSU)

ETU is the most efficient of the three processes since it is closest to the full resonance interaction between the absorbed photon and the absorbing medium.[^136^, ^137^] In Figure 4 A, energy transfer from a directly excited sensitizer is followed by excited state absorption and upconverted emission from the activator, while in Figure 4 B only the sensitizer absorbs lower energy photons, promoting the activator into its second excited state from which upconversion emission occurs in **two** successive energy transfer steps. If sensitizer and activator units are very similar, they can undergo cross‐relaxation photon upconversion (see Figure 4 C).

TTAU commonly features inorganic NCs that are coupled to organic molecules.[^8^, ^9^, ^139^] NCs as well as organic molecules can act as sensitizers undergoing triplet energy transfer (TET) to the activator. The process typically is quite fast and efficient, lying within the range of ps.[^26^, ^31^, ^39^] However, it can also take up to several μs before long‐lived triplet‐states are generated,^[140]^ which are needed to accumulate enough excited triplets for efficient (emissive) triplet‐triplet annihilation. Here, one organic molecule is further excited to a state from which emission takes place, while the other returns to its ground state (see Figure 4).^[139]^ Slow TET rates can be increased to tens of ns by reducing the pristine ligand length of the inorganic NCs to enhance the TTAU. However, the effect is counteracted by increasing dielectric screening when NCs carry very short ligands and are packed tightly, still severely limiting the **overall** speed of TTAU in comparison to ETU or CSU.^[141]^ CSU has also been studied in purely organic binary chromophore mixtures and called cooperative energy pooling here.^[142]^

CSU in comparison to TTAU is considerably faster. Here, for example in a COIN system, two inorganic NCs coupled to organic π‐systems act as sensitizers that are directly photoexcited by lower energy photons. Two excited NCs undergo cooperative sensitization (since at least two sensitizer units participate, the process is called cooperative), followed by an energy transfer between two excited NCs, which in turn leads to charge transfer to an excited state of the coupled molecule from which upconverted photons are emitted. The charge transfer in CSU occurs within a range of tens to hundreds of ps, outpacing the TTAU process by at least one order of magnitude.^[13]^

Note, that a distinction between ETU and CSU is often complicated and upconverting systems can involve all three processes.^[137]^

PU is very efficient in solution,[^30^, ^143^] but up to now there have been only a few solid‐state examples of deposited films showing PU efficiencies in the range of ≈1.5 %,^[139]^ and one recent COIN thin film example exhibiting a PU efficiency of 13 %.^[13]^ Factors that influence the PU efficiency in COIN include the proximity of the sensitizer and acceptor, the binding mode of the molecules to the NC surface and the coupling across the organic/inorganic interface.[^7^, ^25^, ^30^, ^40^]

Thus, concepts for further advancing PU rates and efficiencies include, for example, the utilization of ultrafast hot‐electron mediated PU of plasmonic (nano)structures, which could be coupled to semiconducting NCs or organic π‐systems for efficient emission.^[144]^ Lead‐halide perovskite‐sensitized TTAU is efficient (≈3 %) in solid‐state devices due to the fast transfer of single charge carriers instead of bound triplet excitons/states.^[145]^ Another highly promising concept includes combining ultrathin 2D semiconductors with organic π‐systems to yield 2D COINs. Many organic π‐systems are polyaromatic flat‐on arrangement systems, which should be efficiently coupled chemically and electronically to the 2D semiconductors. Strong exciton binding energies and fast singlet to triplet exciton transfer in 2D semiconductors on the other hand hold high potential for fast and efficient PU in 2D COINs.[^11^, ^48^, ^66^]

### 4.4 Hot charge carrier generation and transfer

After LSPR excitation in plasmonic nanostructures, electron movement in the metal is quickly damped by electron scattering associated with dissipation. Consequently, the excited plasmon mode is lossy and fast decaying (femtosecond range).[^146^, ^147^] Recent advances in photovoltaics^[83]^ and photocatalysis^[148]^ are based on these absorption processes in metals.

In the following section, we thus focus on the mechanism of hot carrier generation by light adsorption in plasmonic metal nanostructures and their transfer to other materials such as semiconductor solids or adsorbates. In addition, we address the requirements for efficient transfer of hot charge carriers and derive design criteria for nanostructures.

**Figure 5:**
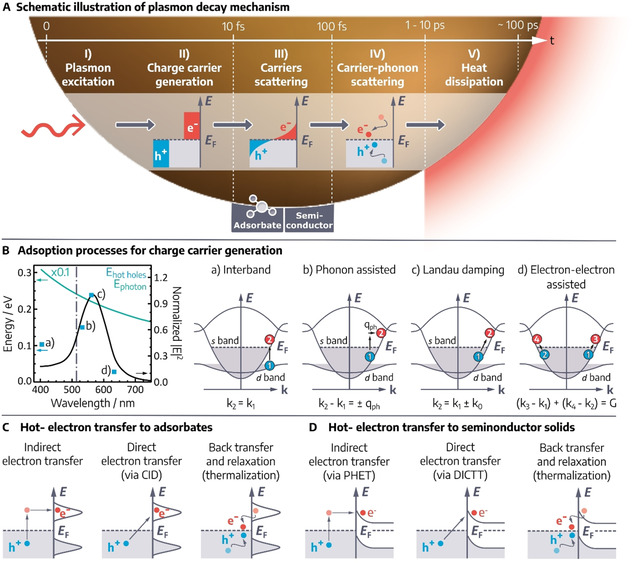
Hot charge carrier generation and transfer. (A) Schematic illustration of the plasmon excitation in nobel nanoparticles and subsequent relaxation process mechanism in dependence of time. (B) Wavelength dependent adsorption mechanism resulting in excited charge carriers of different energy. Left graph includes the hot holes energy contribution (blue squares, left axis), the energy of the incident photons (blue solid curve, left axis) and the square of the electric field |*E*|^2^ inside the NP (black curve, right axis) as function of the wavelength. The vertical dashed line indicates the threshold wavelength for interband excitations. The four band diagrams illustrate the predominant adsorption mechanism in dependency of the different wavelength. Adapted with permission from Ref. [151]. Copyright (2019) American Chemical Society (C,D) Mechanistic description of plasmon‐induced hot carrier transfer/back‐transfer processes in metal/adsorbate, and metal/ semiconductor systems. Adapted with permission from Ref. [152]. Copyright (2018) American Chemical Society.

The relaxation process after LSPR excitation can be either radiative by re‐emitting photons or non‐radiative (see Figure 5 A), resulting in energy transfer and excitation of hot electron‐hole pairs.[^149^, ^150^] For subsequent transfer to other materials, two main criteria have to be met: On the one hand, charge carriers need a minimal critical energy and matching momentum for the cross‐over and on the other hand, the transfer has to take place during charge carriers’ lifetime.

The critical energy, is thereby defined as the energy difference between the Fermi level of the metal and either the conduction band of the semiconductor (Schottky junction)^[153]^ or the LUMO of the molecule (activation energy).^[152]^ Dependent on the excitation wavelength four different adsorption mechanisms can occur during the decay (see Figure 5 B). Each of the mechanisms leads to charge carriers with different energy and mobility. Neither interband absorption nor electron‐scattering assisted absorption (see Figure 5 B.a and 5 B.b) lead to electrons with sufficient energy to cross the energy barrier. In contrast, phonon & defect assisted absorption and Landau damping (see Figure 5 B.c and 5 B.d) are able to generate highly energetic (“hot”) electrons. Landau damping is the preferred mechanism for plasmon induced charge transfer processes, as the hot charge carriers are generated directly on the metal surface, making them more easily accessible.^[146]^ In order to achieve an absorption dominated by Landau damping, it is also recommended to use nanostructures with dimensions below the electrons mean free path length.[^146^, ^151^] For gold and silver, this criterion applies to particles with a diameter smaller than 30 nm. In this size range little scattering is to be expected and interactions are preferentially located at the surface.^[154]^

Besides the excitation wavelength and particle size, the particle shape also affects the yield of hot charge carriers. Experimental and theoretical studies of Au/TiO2 structures have shown that the catalytic efficiency (hot charge carrier generation) is strongly dependent on the EM field enhancement of the plasmonic component.^[155]^ Compared to spherical and rod‐shaped Au nanoparticles, nanostars exhibit the highest photocatalytic efficiency, due to the most intense EM field enhancement around its tips.^[76]^

Geometry as well has a major impact on the required match of momentum:^[156]^ After their generation, the charge carriers have a uniform spatial distribution of momenta.^[157]^ As most of the metal‐semiconductor interfaces, for example, consist of a single planar junction, the charge separation is strongly limited due to this momentum distribution. Only those electrons that have both, sufficient kinetic energy and the right momentum direction (perpendicular to the materials interface) can transfer to the semiconductor. Thus, most of the hot carriers are reflected at the interface, leading to poor efficiency of hot‐electron extraction.[^156^, ^157^] One method to improve the charge transfer yield is the introduction of a 3D instead of 1D Schottky barrier (increase in contact area) by partially embedding the metallic nanostructures in the semiconductor layer.^[157]^

Concerning life‐times, as depicted in Figure 5 A, after plasmon excitation (<10 fs),^[147]^ the initially formed energetic charge carriers rapidly relax and redistribute their energy through electron‐electron scattering resulting in isotropic energy distribution (Fermi‐ Dirac distribution).^[158]^ Within this time frame (<100 fs), either the hot charge carriers are emitted into another material, or they transfer their energy to the lattice through electron‐phonon collision (1–10 ps), elevating the lattice temperature. Heat is then dissipated from the nanoparticle to the environment by phonon‐phonon scattering (100 ps).^[159]^

As shown by Wu et al., an effective transfer rate can be achieved within the given time (<100 fs) by ensuring a strong electronic interaction between the metal structure and a semiconductor material.^[160]^ Initially, it was assumed that the charge separation takes place via a plasmon‐induced hot electron transfer mechanism (PHET) in which the charge carriers are first excited in the metal and then transferred to the inorganic semiconductor solid.^[160]^ According to the decay mechanism (Figure 5 A+D), this process is in direct competition with the relaxation of electrons through electron‐electron scattering or electron‐phonon scattering. Thus, for high efficiency, the transmission has to take place within 100 fs, which is difficult to realize for a classical PHET.^[161]^ Another approach is the direct metal‐to‐semiconductor interfacial charge‐transfer transition (DICTT).^[160]^ In this case, the plasmonic particle serves as a light absorber, and the strong coupling of the metal and semiconductor energy levels leads to a direct generation of an electron in the semiconductor material and a hole in the metal.[^160^, ^162^] However, this interface transition is very weak at the nanoscale.^[160]^ Thus, ideally, a combination of both mechanisms is desirable to create an effective photo‐induced charge separation pathway.

Similar to inorganic semiconductor materials, two mechanisms for hot charge transfer to adsorbed molecules, such as those in section 3, are known (see Figure 5 C). In the classical indirect transfer mechanism, hot electrons are first generated in the metal and then transferred to the LUMO of the adsorbed molecule.^[151]^ Although this process is generally regarded as the dominant mechanism, recent studies suggest a second mechanism via a direct electron transfer pathway resulting from chemical interfacial damping (CID).^[163]^ Experimental observations have shown that a material that is chemically bound to a plasmonic nanoparticle can lead to the acceleration of the plasmon dephasing and thus CID.^[164]^ In this case, plasmon decay can occur by directly generating hot electrons in the electron accepting states on the adsorbate, leaving a hot hole on the metal. In comparison, direct electron transfer is considered to be more efficient than indirect electron transfer, as the latter suffers from significant losses due to electron‐electron scattering.^[152]^ Moreover, experimental evidence shows that CID by adsorbates is able to retard the thermalization process of hot charge carriers (picosecond regime) by the repeated back and forth transfer of hot electrons between the metal and the adsorbates.^[165]^ Some factors need to be taken into account in order to increase efficiency by CID.^[166]^ In contrast to the indirect transfer mechanism, the direct electron transfer requires not only an orbital overlap, but also strong hybridization between the metallic nanoparticle surface and the adsorbate.^[152]^ Furthermore, CID only takes place when the LSPR exactly matches the energy gap of the HOMO–LUMO transition of the hybridized state. Consequently, the direct transfer mechanism is strongly dependent on the LSPR wavelength.^[152]^

As the probability of hot charge carriers generation is dependent on the EM field enhancement, the use of nanogap‐mediated surface plasmon coupling provides another way to enhance the EM field by the targeted formation of hot spots between particles.^[167]^ The intensity of the coupling effect and the EM field enhancement, thereby, strongly depends on the particle distance.^[81]^ Thus, control over the particle spacing and the introduction of ordered particle assemblies is necessary to make efficient use of the coupling effects. In contrast to disordered particle arrangements, hexagonal packed particle layers exhibit a homogeneous distribution of hot‐spots.^[168]^ Plasmonic 1D assemblies in direct contact with semiconductor solids are of potential interest to improve hot charge separation, by supporting both, local plasmon resonances and waveguide modes.^[169]^

With proper adjustment of the particle size, the distance between the particle lines and the waveguide thickness, strong coupling between the plasmonic and photonic resonance can be achieved (details see Ref. [168]), resulting in hybridized modes which are characterized by a strong suppression of radiative damping.^[170]^ Therefore, the strong interaction between plasmonic 1D structures and a semiconductor solids allows the improvement of the generation and injection performance of hot electrons.[^169^, ^170^, ^171^]

## 5 Characterization and Device Fabrication Tools

### 5.1 Time‐resolved spectroscopy tools

Time‐resolved spectroscopy (e.g. in the optical range of UV/Vis to NIR, 3.5–0.8 eV) is an excellent tool for studying photophysics on an ultrafast time scale in the materials and material combinations mentioned above (typically from fs over ps to ns). This is important for resolving the underlying photoexcitation processes, including charge carrier generation, migration and recombination dynamics as well as energy transfer and change of charge carrier spins in “real time”. Samples to be characterized are typically measured in solution or as thin solid films at ambient temperature (in general measurements at lower and higher temperatures are possible with a cryostat). Thin COIN films exhibit sizes of several hundreds of μm so that a probe beam spot size of <0.5 μm and a pump beam spot size of >1 μm are sufficient to probe the sample accordingly. Multiple sample areas are spectroscopically characterized independently to ensure sufficient statistics. While excellent reviews on ultrafast spectroscopy exist,^[172]^ we will give a brief overview on the techniques considered particularly useful for characterizing ultrafast charge carrier dynamics in COINs.

#### 5.1.1 Time‐resolved photoluminescence spectroscopy (TRPLS)

In TRPLS, the radiative recombination (TR‐photoluminescence, PL) of photoexcited charge carriers (excitons) in samples (e.g. inorganic/organic semiconductors, nanomaterials, hybrid structures, COINs, molecules or plasmonic materials) is spectrally and temporally resolved. In a typical *pump‐probe* experiment, the sample is photoexcited with an optical *pump* pulsetrain of an ultrafast laser source (≈100 fs), while a delayed *probe* pulsetrain enables monitoring of the sample PL at different times after photoexcitation. TRPL measurements with a time resolution down to ps can be conducted with a Streak camera, an ultrafast detector capturing the sample emission.^[173]^

#### 5.1.2 Transient absorption spectroscopy (TAS)

In TAS, samples are likewise photoexcited with a short *pump* pulsetrain, while a delayed *probe* pulsetrain is used to study the evolution of photoexcited charge carrier dynamics at different times after photoexcitation. The temporal and spectroscopic time evolution of the change in absorption of the sample is used to draw conclusions about the origin and fate of the photoexcited charge carriers, as well as their decay dynamics.^[174]^ An advantage of TAS in comparison to TRPLS is its sensitivity to non‐emissive dark states, which naturally cannot be probed by PLS.

Recent investigations of different COINs with TAS have successfully proven chemical and electronic coupling, polaron formation, upconversion by TTA and CSU, ultrafast energy and charge transfer and underline the versatility of the method.[^34^, ^40^, ^43^, ^143^]

### 5.2 Tools for nanocrystal assembly

Colloidal self‐assembly is a thermodynamically driven process in which defined building blocks (e.g. nanocrystals) are spontaneously organized into ordered structures.^[175]^ The self‐assembly process is controlled by the interaction of various attractive (van‐der Waals and Coulomb/double layer forces) and repulsive (Coulomb/double‐layer and steric repulsion) forces, which are defined by the physical and chemical properties of the nanocrystals and the corresponding interface or substrate.^[176]^ For achieving complex/ heterogenous patterns, directed self‐assembly processes are used: External driving forces can be applied by introducing molecular scaffolds (e.g. DNA), templates, electric or magnetic fields.^[177]^

For well‐controlled colloidal self‐assembly both geometry and surface properties are crucial.^[77]^ Dispersity in size and shape leads to disruption of the assembly process and to the formation of defects. colloidal interactions are essential for the self‐assembly process and have to be adapted to the chosen process through appropriate chemical functionalization.

#### 5.2.1 Interface‐mediated nanocrystal assembly

**Figure 6:**
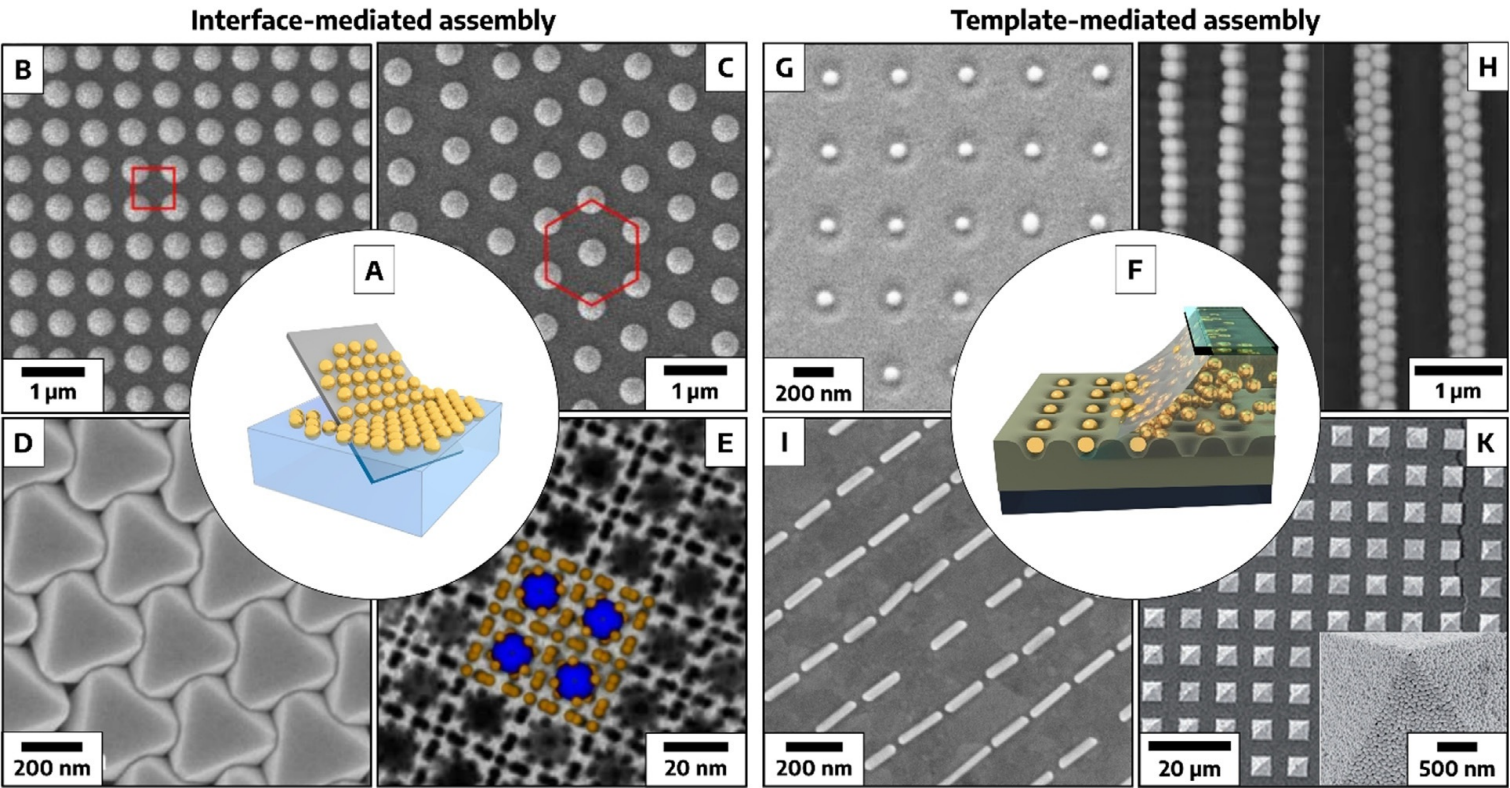
Tools for Nanocrystal Assembly. (A) Schematic illustration of Interface‐mediated assembly. (B,C) Particle monolayers with square or hexagonal Bravais lattice order. Adapted with permission from ref. [192]. Copyright (2019) American Chemical Society. (D) Monolayer of PVP‐functionalized Ag octahedra formed by dip‐up self‐assembly. Adapted with permission from ref. [179] Copyright 2015, Nature Publishing Group. (E) HAADF‐STEM image of NaZn13‐type binary nanocrystal superlattices including a structural model of the [001] projection. Adapted with permission from ref. [14] Copyright 2015, Springer Nature (F) Schematic illustration of template‐assisted assembly of plasmonic nanoparticles and (G) corresponding SEM image of the fabricated 2D plasmonic lattice array. Adapted from ref. [188] Copyright 2019 American Chemical Society. (H) AFM images of selective deposition of 380 nm‐sized colloidal particles on wrinkled films of PAH‐PSS multilayers. Adapted from ref. [193]. Published by The Royal Society of Chemistry (I) SEM image of gold nanorods organized in single lines by wrinkle‐assisted dip coating procedure. Adapted with permission from ref. [194]. Published by The Royal Society of Chemistry (K) Macroscale nanostructured film with pyramids consisting of gold nanoparticle building blocks. Adapted with permission from ref. [195] Copyright 2013, Wiley‐VCH Verlag GmbH & Co. KGaA.

Periodic superstructures of nanocrystals can be achieved via interface‐mediated assembly (examples see Figure 6 A–E). To form densely packed, minimal free‐energy structures, the particles require the ability to adsorb at the interface while maintaining a certain degree of mobility.^[178]^ In interfacial assembly methods such as drop casting, spin‐coating or Langmuir–Blodgett, liquid/liquid and liquid/air interfaces are used to facilitate these criteria. At the interface, the adsorbed nanocrystals are influenced by long‐range attractive and short‐range repulsive interactions. If attractive capillary forces dominate the system, densely packed hexagonal or cubic lattices are formed.^[179]^ Beyond monolayer formation, three‐dimensional NC assemblies with macroscopic long‐range order can be achieved, for example, by gradual solvent destabilization.^[180]^ The narrowing of the NC size and shape distribution enables densely packed structures and stabilizes the reactive surfaces of NCs by minimizing interparticle spacing and maximizing the coordination number. A strong thermodynamic driving force results from this stabilization, enabling the formation of extended superlattices of NCs with long‐range order.^[181]^ Equally important is the prevention of kinetic arrest, which can lead to frequent defects or glass‐like assemblies.^[182]^ In this regard, solvent vapor annealing or slow evaporation and crystallization from solution by the gradual addition of an anti‐solvent have proven to be effective. With these techniques, it is now possible to achieve single‐crystalline superlattices of NCs over areas of ≈100 μm^2^.^[183]^

#### 5.2.2 Template‐mediated nanocrystal assembly

While the interface‐mediated assembly is a simple method for large‐scale assemblies, template‐mediated nanocrystal assembly enables particle arrangements with a wide range of possible array geometries (examples see Figure 6 F–K). The template provides specific binding properties for nanocrystals, which include topographical traps, chemical linkers to form bonds or attractive interactions, and spacers which are based on repulsive interactions. As an example, microcontact printing is used to imprint chemical contrast on a target substrate by transferring or removing certain binding sides. The chemical contrast can consist of charge differences (e.g. electrolytes),^[184]^ hydrophobic contrasts (e.g. silanization)^[185]^ or the targeted use of reactive linking groups.^[186]^ Due to its functionalization, the particles will only attach to the matching surface patterning, when approaching the surface.

Topographic templates are usually fabricated by lithographic methods, like electron‐beam lithography,^[187]^ UV‐interference lithography,[^168^, ^188^] or soft lithography,^[189]^ providing complex structures like periodic lines, elliptical holes/pillars in hexagonal order or square lattice. However, lithographic methods are usually cost‐intensive and time‐consuming, poorly scalable or limited in achievable feature size. An alternative approach to obtain macroscopic templates of cm^2^—area with nano‐sized structure dimensions is the wrinkling of PDMS substrates.^[190]^ As demonstrated, these templates are suitable for directing the self‐assembly of colloidal particles within common assembly techniques such as drop casting, spray coating, spin coating, dip coating or capillary assisted self‐assembly.[^77^, ^191^]

## 6 Fabrication Strategies for Optoelectronic Applications

### 6.1 Strategies for singlet fission with coupled organic‐inorganic nanostructures

As described under 4.1, SF could allow the breaking of the Shockley‐Queisser limit, and implementation with different PV devices was shown to enhance photocurrent in organic and hybrid solar cells.[^12^, ^196^] Sufficient light absorption and a high enough concentration of SF chromophores to harvest most photons is difficult to realize in bilayer structures.^[128]^ COIN structures might allow a high enough chromophore and sufficiently low NC concentration to make practical devices possible. For an efficient SF process, a spatially (and electronically) intimate arrangement is needed to support the bimolecular excimer mechanism. In solutions of organic molecules, this translates to relatively high molecular concentrations,^[197]^ and in dilute solutions, a conscious bridge design allows to study the electronic coupling of dimers. In COIN structures, separating organic chromophore and NC via electronically inactive surface ligands hinders the Dexter‐like transfer process,^[134]^ requiring a spatially unobtrusive or electronically mediating surface ligand, or the direct coupling of the SF chromophore.[^26^, ^128^] For the direct coupling, a carefully chosen anchor group is key: The anchor group should (1) allow the chromophore to stack, (2) create spatial proximity and (3) enable a mechanically stable coupling of NC and organic chromophore, as well as (4) a high surface density of the latter.

Investigations of COIN‐supported SF are very recent, and as yet comprehensive studies on design strategies for anchor groups and suitable ligand arrangements are lacking. A final challenge for application is the transfer into solid state: Here, not only the energetic and spatial alignment of NC and organic chromophore must be tuned, but also the nanomorphology needs detailed control.

### 6.2 Fabrication principles for triplet exciton harvesting with coupled organic‐inorganic nanostructures

We focus here on three applications of triplet harvesting in devices, namely triplet‐triplet annihilation for photon upconversion, spin storage for memory applications and triplet diffusion for photovoltaics. As outlined in section 4.2, the major challenges to be addressed in order to excel in these applications are: A reduction of the interchromophore distance, maximization of the regioregularity of the nanostructure, an increase of the orbital overlap at the hybrid interface and a prevention of intermolecular relaxation as well as the suppression of excimer formation to increase triplet lifetimes. Apart from triplet diffusion, another requirement is establishing a unidirectional triplet transfer, that is, the inhibition of triplet back‐transfer.

Since triplet transfer from the sensitizer to the acceptor is usually the rate limiting step during harvesting (section 4.2), tailoring the triplet transfer rate is of paramount importance for all device applications. For triplet‐triplet annihilation and spin storage, the focus should be on fast and one‐way triplet transfer onto the acceptor, where the spins exhibit long lifetimes. Therefore, a large negative energy offset between the triplet state of the sensitizer vs. that of the acceptor is important. Based on the criteria for TET following Marcus theory in section 4.2, a small effective mass for the hole and electron in the sensitizer as well as low reorganization energy are also desirable. These criteria can be met by decreasing the NC size and maximizing the quantum confinement.^[25]^ In contrast, optimizing triplet diffusion for photovoltaics requires forward and back transfer of triplets between the sensitizer and the acceptor to be equally efficient in order to achieve a large triplet diffusion length. Therefore, an energy offset as well as substantial differences in the reorganization energy or carrier effective masses should be avoided. Here, a high degree of regioregularity is key.

COINs with large areas of structural periodicities and long‐range order, such as those depicted in Figure 6, benefit from the absence of grain boundary scattering, uniform orientation of molecular orbitals at the hybrid interface and the formation of closed‐packed structures with minimized interchromophore distances. While it is currently not clear which superlattice structure or NC orientation should be preferred for triplet diffusion, tailoring the regioregularity in conjunction with a time‐resolved spectroscopic feedback mechanism should result in significant improvements. Introducing molecular π‐systems to an ordered array of NCs without compromising its regioregularity can either be achieved by evaporation of a molecular layer on top of the superlattice^[139]^ or by ligand exchange of the NCs with the organic π‐system.^[198]^ The disadvantage of the former method is the limited mixing of the inorganic and organic constituents, while the latter can lead to cracks and other defects in the superlattice if the length of the organic π‐system is substantially different from that of the native NC ligand. To address this challenge, interface‐mediated ligand‐exchange (section 5.2.1) of the superlattice on a liquid substrate has proven to be advantageous, since floatation of the NCs at the liquid/gas interface renders the superlattice more tolerant towards geometrical changes.^[199]^ Alternatively, template‐mediated assembly (section 5.2.2) provides a rigid framework for the superlattice such that regioregularity is also maintained after functionalization of the NCs with organic surface molecules.

Optimizing the orbital overlap is more complex as this depends to a large degree on the exact binding motif of the molecules to the NC surface, which is unclear in most cases. For metal phthalocyanines, optical spectroscopy has suggested that the molecular π‐system forms small stacks of H‐aggregates, which separate adjacent NCs.^[13]^ X‐ray scattering found interparticle distances of precisely one molecular length, suggesting that the π‐cloud of the phthalocyanine stacks is oriented perpendicular to the binding axis between two NCs.^[200]^ It seems likely that this highly specialized scenario is not of a universal nature and that other binding motifs between NCs and molecular π‐systems are possible (see section 3.2). However, to systematically exploit this and other structural diversities in hybrid nanostructures for triplet harvesting, a better understanding of an optimal binding geometry is needed. A notable first step in this direction was a systematic study of bis‐pyridine anthracene derivatives with different substitution patterns.^[40]^

Fast radiative or non‐radiative recombination of triplets are among the most important challenges for devices, which rely on long lifetimes and diffusion lengths for efficient triplet harvesting. To overcome the inherent “spin‐mixing” behavior of NCs, operating at low temperature (≈10 K), where the triplet state is stabilized will be mandatory for applications requiring a high spin purity and maximized lifetimes, such as spin memories. Another factor with deleterious consequences for the spin purity are dangling bonds and other surface defects, which can invoke spin flipping due to the interaction with the unpaired electrons in dangling bonds and are, thus, to be avoided.^[21]^ The most common strategies involve the design of surface molecules with the right electronic structure to remove trap states from the band gap, the growth of an epitaxial shell onto the NC core or filling the gaps within NC solids by an inorganic, macroscopic matrix, all of which have been outlined in section 1.1. Finally, it should be emphasized that the stability of triplets is hugely affected by the presence of oxygen, such that efficient triplet harvesting will benefit strongly from operation under inert conditions, for instance by encapsulation.

**Figure 7:**
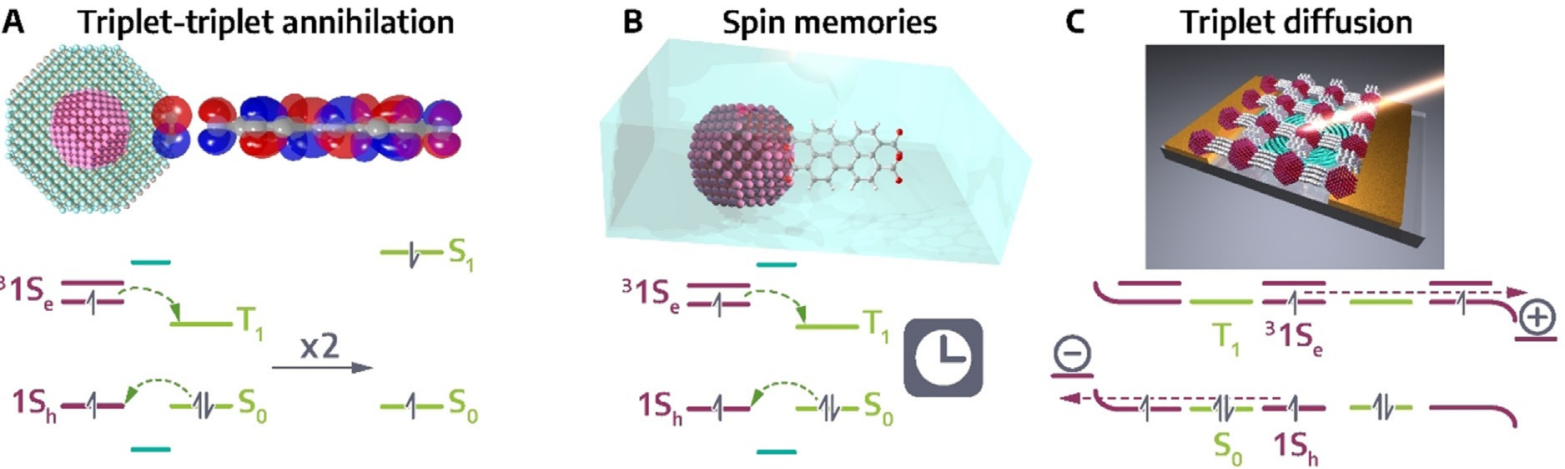
Device outlines for triplet harvesting devices. A) Triplet‐triplet annihilation, B) spin memories and C) triplet diffusion for photovoltaics.

Figure 7 suggests architectures for all three triplet harvesting devices discussed here: 1) A material optimized for triplet‐triplet annihilation (Figure 7 A) should comprise of QDs or two‐dimensional semiconductors with large quantum confinement, a thin, passivating shell to remove surface defects and a high‐lying triplet state. The organic component should provide a low‐lying triplet state with relatively long lifetime, a singlet state of less than twice the triplet energy and a binding motif to the inorganic surface which enables large overlap between the organic and inorganic frontier orbitals. This requires a high degree of regioregularity to ensure that all interfaces in a large ensemble of COINs are identical in this respect. 2) For spin memory applications (Figure 7 B), we envision a hybrid spin memory, in which spin is generated in the NC and stored in the surface molecule. Similar to the considerations for triplet‐triplet annihilation, such a system should entail unidirectional triplet transfer without significant back‐transfer. The singlet state in the organic component should be more than twice as energetic to prevent triplet‐triplet annihilation. Since stability is the key property and only isolated COINs are needed, an encapsulation strategy appears preferential over the growth of a thin shell.^[201]^ This will also limit the deleterious exposure to oxygen and restrict vibrational motion of the organic molecules to extend triplet lifetimes. 3) Triplet diffusion needs balanced triplet transfer and back‐transfer at the hybrid interface to prevent a trapping of triplets (Figure 7 C). This is enabled by selecting inorganic QDs or 2D nanostructures with ground state and triplet energies that are resonant with those in the organic components. Since the triplet mobility is key in these devices, wide band gap shells or matrices should be avoided, and surface defects must be managed by virtue of suitable functional groups of the organic components (section 3.1). To prevent scattering of triplets, regioregularity with near‐single crystalline superlattices, uniform molecular orientation and orbital overlap at all interfaces as well as a constant interchromophore distance are of highest importance.

### 6.3 Assembly of coupled organic‐inorganic nanostructures for photon upconversion

As has been described in the previous sections, close contact between the organic‐inorganic moieties of a COIN is the key for efficient coupling and PU. If ETU is favored, energy level alignment of the sensitizers has to be selected such that their (first) excited state is close to the first excited state of the activator to enable efficient Förster resonance energy transfer (FRET). However, a spectral overlap of the acceptor emission and the sensitizer absorption could lead to downconversion from the sensitizer. This has to be avoided.^[142]^ TTAU is efficient in COINs, however, intersystem crossing and efficient TET requires significant synthetic tailoring and TTAU speed is limited by the accumulation of sufficient triplet states for annihilation to occur. Additionally, the quenching of triplet‐states by oxygen might hinder solid‐state applications under environmental conditions without encapsulation. Generally, CSU seems to be the preferred mechanism for utilizing low‐loss solid‐state thin‐film based PU. Requirements for CSU include the coupling of two excited sensitizers with the two‐photon absorption tensor/cross section of the acceptor system, leading to simultaneous and ultrafast excitation and subsequent PU from the activator. For further boosting CSU efficiencies, sequential photon absorption in sensitizers should be prevented.[^142^, ^202^] First results on CSU are very promising in COINs and have been shown for coupled lead sulfide zinc phthalocyanine structures.^[13]^ Improving the PU emission quantum yield in these structures could be reached by for example, coupling more strongly emitting organic π‐systems to the NCs. Finally, PU depends on the relative orientation of the sensitizer and activator units to each other.^[202]^ In respect to this, 2D COINs comprising of flat ultrathin semiconductors tethered to organic π‐systems offer a highly interesting new degree of freedom.

### 6.4 Realization of hot charge carrier transfer with coupled organic‐inorganic nanostructures

As described in section 3.5, plasmonic nanostructures are able to convert absorbed light into electrical energy by the excitation of hot charge carriers. Thus, the plasmon‐induced hot carrier generation is a promising tool for energy conversion processes in photovoltaics or photochemistry.^[203]^ In addition to the further development of the commercially most used Si‐based solar cell, the development and improvement of flexible and cost‐effective organic solar cell systems has become a central issue in the field of renewable energy sources. In the following section, we will focus on solar cell improvement through the use of plasmonic induced hot‐charge generation.

**Figure 8:**
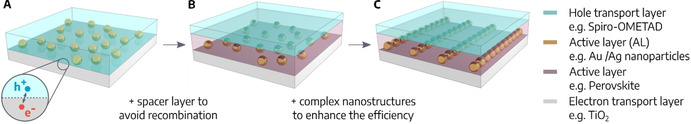
Realization of hot‐charge carrier transfer processes in organic solar cells. (A) Schematic depiction of a plasmonic organic solar cell including three layers: TiO_2_ as electron transporting layer, plasmonic nanoparticles as active layer and Spiro‐OMETAD as hole transporting layer from bottom to top. Architecture of a plasmonic enhanced perovskite solar cell with perovskite and random distributed nanoparticles (B) or ordered 1D nanostructures (C) serving as active layer.

In the simplest case, the plasmonic nanostructure is implemented as an active layer in the solar cell (see Figure 8).^[83]^ Consequently, the selection of the nanoparticle system (material, shape and size) and the type of nanostructure are decisive for the efficient generation and transfer of hot charge carriers (for details see ssection 3.5). In addition, the material of the semiconductor and its interaction with the plasmonic component play an important role in the separation performance. In order to avoid a later recombination of hot carriers due to charge imbalance and to obtain an electric current, a corresponding electron donor solution or a hole conducting material is required. However, it has been shown that direct contact between the hole‐conducting layer and the semiconductor material can lead to harmful carrier recombination and thus to a reduction in efficiency.^[204]^ Thus, also the architecture and the materials of choice are important for the construction of a plasmonic organic solar cell.

At the current state of research, Au and Ag are the best‐investigated and most frequently used metals for plasmonic components, since they exhibit relatively low losses in the visible spectral range.^[205]^ However, Cu and Al have also recently gained importance. In contrast to Au and Ag, Al is an abundant and relatively cheap metal. It also exhibits a more positive work function than Au and Ag, resulting in a smaller metal‐semiconductor Schottky barrier and thus an increased probability of hot carrier transfer.^[171]^ In addition, efficient charge separation also depends on the semiconductor material. TiO2 has been shown to be a well‐suited material as it has a wide band gap and excellent electron acceptability due to the high density of states in its conduction band.^[206]^

When considering the use of electron donor solutions or hole transport layers for charge balancing, it was shown that the use of liquid cells leads to a higher degree of efficiency, but is impractical for photovoltaics due to a lack of stability.[^149^, ^207^] In recent years, several plasmonic solid‐state solar cell structures with organic or inorganic hole transport layers have been proposed. An overview of the research progress in this field can be found in the article by A. Furube and S. Hashimoto.^[207]^ In the following, we will focus on solar cells using Spiro‐OMeTAD, one of the most successful hole conductor materials,^[208]^ and, in our opinion, a promising material in this context. Reineck et al. introduced a solid‐state cell in which Au and Ag nanoparticles serve as the active layer and Spiro‐OMeTAD as the hole transport material, achieving an IPCE of 4.9 and 3.8 %, respectively.^[209]^ They also showed that the efficiency of this solar cell strongly depends on the particle size used.^[210]^ However, a disadvantage of this structure (see Figure 8 A) is the direct contact between the semiconductor and the hole transport layer, as undesired recombination effects occur. By introducing a further layer using the architecture of perovskite solar cells (see Figure 8 B), significantly higher efficiencies can be achieved. Mali et al. presented the implementation of Au‐TiO2 systems in perovskite solar cells with an efficiency of 14 %.^[211]^ Recently published results show that the optimization of the metal‐semiconductor system and the use of more complex nanoparticle systems (see Figure 8 C) can achieve a power conversion efficiency of 19.16 %.^[212]^

Another promising approach to increasing efficiency could be the integration of ordered lattice structures (see section 3.5) in perovskite solar cells. By coupling plasmon hot spots with photonic lattice effects, the absorption of the solar cell and the generation of hot carriers can be significantly increased. In addition, the direct coupling between the plasmonic component and the semiconductor material allows better charge separation.

## 7 Summary and Outlook

We began this review with a hypothesis originating from the 1980s that biphasic nanocomposites can exhibit emergent properties which are more than the sum of the individual contributions if the effect of the interface becomes predominant. The large body of work discussed here shows that maximizing the interfacial area and controlling electronic coupling in such hybrid nanocomposites indeed fulfills this promise. Enabling charge or energy transfer across the interface enhances the importance of the phase boundary, providing additional optoelectronic functionalities. Tailoring the transfer direction exploits the vast differences between inorganic and organic nanostructures in terms of their behavior as sensitizer or acceptor agents during the transfer. The largest advantages of hybrid nanocomposites over single‐phase materials arise if two components with excellent acceptor/poor sensitizer properties and *vice versa* are combined to a highly asymmetric interface. Utilizing this asymmetry in the preferred direction enables several optoelectronic applications with high efficiency, of which we have specifically highlighted photon upconversion, singlet fission, triplet exciton and hot electron harvesting. Additional opportunities which we have omitted due to limitations of space include light emitting diodes and photochemical reactions.

The opportunities arising from coupled organic‐inorganic nanostructures have become possible largely due to the development of chemical strategies to tailor the interfaces in the composites. This includes the surface chemistry of the nanostructures, an understanding of the correlation between it and the electronic structure as well as the development of methods to monitor this correlation. There have also been large improvements in controlling the micro‐ and macroscopic structure of the composites, for instance by self‐assembly and templating. Perhaps the largest challenge for chemists in the future is understanding and tailoring the structure and binding situation at the hybrid interface itself. Very limited knowledge exists to date about the precise binding motif, the orientation of the organic entity to the surface of the nanostructure and possible polymorphs and variances for a given combination. Considering the large dependence of the optoelectronic effects discussed here on orbital overlap, regioregularity and interchromophoric distance, it is highly likely that excelling in controlling the structure at the interface provides room for substantial further device improvements.

## Conflict of interest

The authors declare no conflict of interest.

## Biographical Information

*Anja Maria Steiner received her Master's degree in 2015 from the University of Bayreuth, where she studied Polymer Science. She is currently a Ph.D. student at the Leibniz‐Institut für Polymerforschung Dresden under supervision of Prof. Dr. Andreas Fery. Her research interests mainly focus on the synthesis, assembly and characterization of nanoparticle systems*.

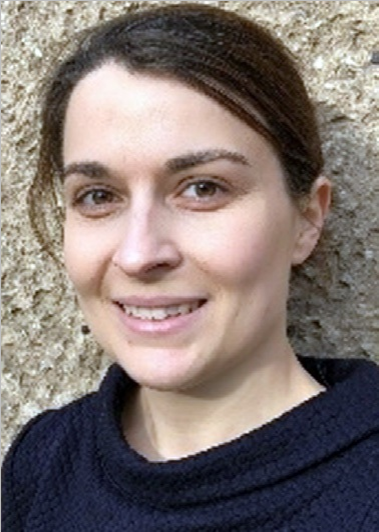

## Biographical Information

*Franziska Lissel is a TUD Young Investigator and a Liebig Fellow of the Chemical Industry Fund (FCI) heading the Functional Electronic Materials group within the Leibniz‐Institute of Polymer Research. Her group investigates the covalent introduction of redox‐active metalcenters into conjugated polymer backbones, the development of polymeric MALDI matrices, new concepts for stretchable polymer electronics, and molecular switches. Previously, she was associated with Prof. Z. Bao (Stanford), and obtained her Ph.D. with Prof. H. Berke (Zürich 2014)*.

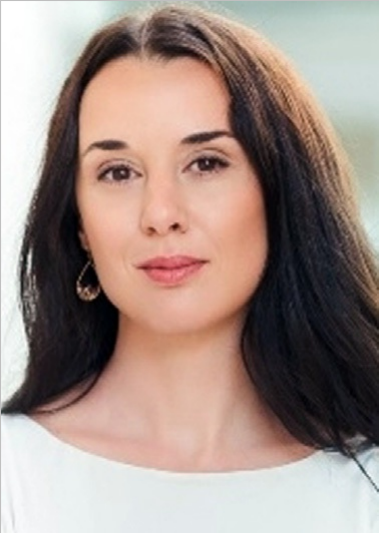

## Biographical Information

*Andreas Fery did his PhD at the MPI for Colloids and Interfaces (MPIKG)/Potsdam University in 2000. After a post‐doc at Institute Curie, he became group leader MPIKG. In 2007 he joined Bayreuth University as professor. Since 2015 he is head of the institute for Physical Chemistry/Polymer Physics at the Leibniz Institut für Polymerforschung Dresden. He is interested in colloid and interface science with a focus on colloidal interactions and colloidal self‐assembly of functional materials such as plasmonic metal surfaces*.

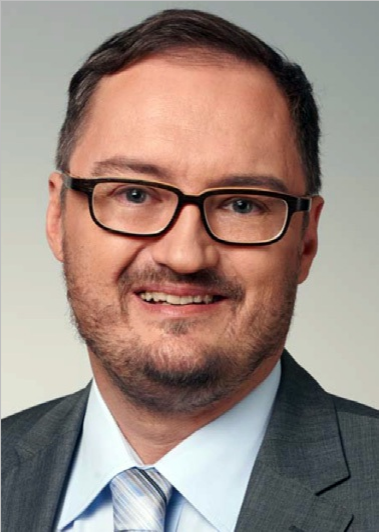

## Biographical Information

*Jannika Lauth obtained her Ph.D. in 2014 at the University of Hamburg with Prof. H. Weller. She joined TU Delft as a postdoctoral fellow with Prof. L.D.A. Siebbeles working on ultrafast laser spectroscopic methods. Since 2019 she is a research group leader at the Physical Chemistry and Electrochemistry Institute of the Leibniz University of Hannover. Her group is combining colloidal synthesis methods (for 2D semiconductors) with assessing the materials’ potential for optoelectronics by ultrafast transient absorption spectroscopy*.

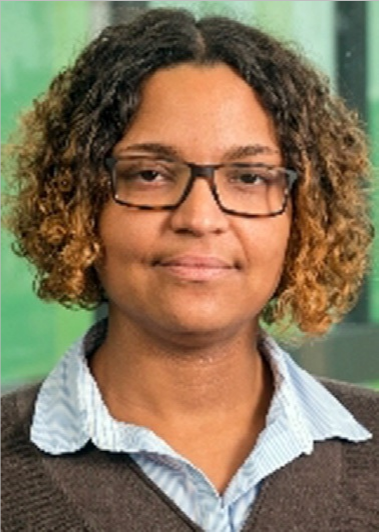

## Biographical Information

*Marcus Scheele obtained his PhD at the University of Hamburg in 2011. After a postdoctoral visit at UC Berkeley, he led an Emmy Noether group at the University of Tuebingen and was appointed as a Heisenberg Professor for Physical Chemistry in 2020. His group investigates the physical chemistry of hybrid semiconductor mesocrystals and their application for fast optical data communication*.